# Design, synthesis, and unraveling the antibacterial and antibiofilm potential of 2-azidobenzothiazoles: insights from a comprehensive *in vitro* study

**DOI:** 10.3389/fchem.2023.1264747

**Published:** 2023-09-07

**Authors:** Tanzeela Qadir, Saadat A. Kanth, Mohammad Aasif, Abdalla N. Fadul, Gulam N. Yatoo, Kailash Jangid, Mushtaq A. Mir, Wajahat A. Shah, Praveen K. Sharma

**Affiliations:** ^1^ Department of Chemistry, School of Chemical Engineering and Physical Sciences, Lovely Professional University, Phagwara, India; ^2^ Centre of Research for Development and P.G Programme in Microbiology, School of Biological Sciences, University of Kashmir, Srinagar, Jammu and Kashmir, India; ^3^ Department of Chemistry, National Institute of Technology, Srinagar, Jammu and Kashmir, India; ^4^ Department of Clinical Laboratory Sciences, College of Applied Medical Science, King Khalid University, Abha, Saudi Arabia; ^5^ Department of Chemistry, Central University of Punjab, Bathinda, Punjab, India; ^6^ Department of Bio-Resources, Amar Singh College Campus, Cluster University Srinagar, Srinagar, Jammu and Kashmir, India; ^7^ Laboratory of Natural Product and Designing Organic Synthesis, Department of Chemistry, University of Kashmir, Srinagar, Jammu and Kashmir, India

**Keywords:** synthesis, 2-azidobenzothiazoles, antibacterial activity, biofilm inhibition assay, cytotoxicity assay, benzothiazoles

## Abstract

The present study reports the synthesis of 2-azidobenzothiazoles from substituted 2-aminobenzothiazoles using sodium nitrite and sodium azide under mild conditions. All the synthesized compounds were examined for their antibacterial activity against Gram (+) bacteria, *Staphylococcus aureus* (ATCC 25923), *Enterococcus faecalis* (ATCC 51299), *Bacillus cereus* (ATCC 10876) and Gram (−) bacteria, *Escherichia coli* (ATCC 10536), *Pseudomonas aeruginosa* (ATCC 10145), *Klebsiella pneumonia* (ATCC BAA-2146)and clinical isolates of Gram (+) Methicillin Resistant *S. aureus* (MRSA) and Multi Drug Resistant *E. coli*. The Minimum Inhibitory Concentration (MIC) and Minimum Bactericidal Concentration (MBC) values by broth dilution method revealed that compound 2d exhibited significant antibacterial potential against *E. faecalis* and *S. aureus* with MIC of 8 μg/mL, while other synthesized compounds had only moderate effects against all the tested species. The compound significantly inhibited the biofilm formation of the bacterial strains below its MIC. The selective cytotoxicity of Compound **2d** towards bacterial cells was evidenced on extended exposure of Human Embryonic Kidney-293 cell line to higher concentrations of the compound. Hence, the present study confirmed that compound **2d** can be a potential drug candidate for future development as an antibacterial drug.

## 1 Introduction

Aromatic azides are most frequently used as reagents for the photoaffinity labeling of biomolecules despite their unusual uses in synthetic organic chemistry. A relatively small number of transformations are used to synthesize aryl azides (Nicholls et al., 1991; Scriven and Turnbull, 1998; Ali et al., 2014; Lima et al., 2015; Faraji et al., 2017; De la Torre et al., 2018; Almehmadi et al., 2021; Zhilitskaya et al., 2021). This chemistry, while helpful for relatively straightforward ligands, is not necessarily permissive to additional functional groups. It has been investigated to use other techniques, such as p-tosylazide reacting with lithium reagents or aryl Grignard made from the appropriate aryl halides (Tariq et al., 2018). Similar to this, it has been demonstrated that *p*-tosylazide can react with aryl amide salts to produce the required azides. The general application of this transformation has been constrained by somewhat harsh conditions (Fischer and Anselme, 1967). In contrast to aromatic azides, aliphatic azides can be made using a variety of ways. A high-yielding reaction with triflyl azide can easily transform aliphatic amines into the corresponding azides, apart from simple substitution reactions utilizing the azide ion and different electrophiles (TfN_3_) (Wu et al., 2004). This reaction, recently popularized by Wong, has drawn a lot of attention and found several uses (Wang et al., 2003; Siddiki et al., 2013). It has been discovered that aryl azides can be produced from organoboron compounds employing a copper (Ⅱ) catalyst (Nomiya et al., 2000). The diazotization of [ArN_2_] [BF_4_] salts immobilized in [BMIM][PF_6_] ionic liquid is an effective way to produce azido-derivatives (Artyushin et al., 2008). However, only a small number of transformations are used in the synthesis of aryl azides (Warmuth and Makowiec, 2005). They are frequently made from the equivalent amines using the diazonium salts of those amines (Yamazaki et al., 2005). Depending on whether or not there are incompatible functional groups present, this could occasionally be an issue. Alternative approaches have been researched, such as interactions between organometallic aryls and p-tosyl azide, which have been studied (derived from the corresponding aryl halide) (Yalcin et al., 1992). More recently, Liu and Tor have successfully prepared aryl azides using Wong’s (TfN_3_) technique (Moorhouse et al., 2006). Even though it works, this approach has several shortcomings. First, the highly reactive Tf_2_O is used. Second, it has been discovered that TfN_3_ is explosive on its own (Kleinhofs et al., 1978). Tertbutyl nitrite (t-BuONO) and NaN_3_ have recently been reported to be used in the synthesis of aromatic azides by Das et al. (Kini et al., 2007). Phenyl hydrazine derivatives can also be used as a starting material in various processes to produce aromatic azides, including (Palmer et al., 1971) which uses Br_2_/PPh_3_, (Burger and Sawhney, 1968), which uses N_2_O_4_/CCl_4_, and (Soni et al., 2010) which uses O_2_/NO. Using diazonium salt and sulfonamide, Dutt et al. described the synthesis of aryl azide; however, a significant drawback of this technique is the production of sulfonic acid as a byproduct (Liu and Tor, 2003).

In click chemistry, organic azides play a key role (Telvekar et al., 2012). Applications for the cycloaddition of organic azides and terminal alkynes are numerous, including combinatorial drug development, and bioconjugation. The synthesis of triazoles begins with organic azides, one type of 1,3-dipole that is particularly significant (Kolb and Sharpless, 2003). These heterocyclic derivatives have important uses in chemical biology (Siddiki et al., 2013), pharmaceuticals, and material science (Faraji et al., 2017), among others. Additionally, several benzothiazole analogs bearing triazole moiety have been investigated as potential anticancer (Katritzky and Rachwal, 2010; Almehmadi et al., 2021), anti-inflammatory, anti-nociceptive, FGFR1 inhibitors, analgesic and antimicrobial agents (Keri et al., 2015). Fluconazole, other antifungal medications, voriconazole, and albaconazole all include 1,2,4-triazole. However, no commercially available medications include the 1,2,3-triazole ring. Sincere efforts have been made to incorporate 1,2,3-triazole into currently available medications, further study is required to identify the lead molecule (Shafi et al., 2012; Ali et al., 2017; Ashour et al., 2020). Moreover, benzothiazole analogs serve as an important scaffold for further molecular research to produce novel compounds (Weekes and Westwell, 2009; Lauria et al., 2014). Hence, we were intrigued by the opportunity to design new compounds that would be very useful in click chemistry, for which ***Carolyn R. Bertozzi***, ***Morten Meldal***, and **K. *Barry Sharpless*** got the ***Noble Prize in 2022***, and would be utilized for the formation of biologically significant scaffolds. Recently, Singh et al. synthesized triazoles derived from benzothiazole as promising antimicrobial agents and various substituted aryl azides were reacted with dialkyne substituted 2-aminobenzothiazole to generate desired compounds by click chemistry. The compounds showed maximum potency against all Gram (+)/Gram (−) bacterial strains with a MIC value of 3.12 μg/mL, which is two times more active as compared to the standard drug ciprofloxacin (MIC 6.25 μg/mL) (Singh et al., 2013). Similarly, Jakopec et al. synthesized and evaluated the cytotoxicity activity of 1,2,3-triazoles of benzothiazole derivatives (Jakopec et al., 2022). Both methodologies utilized dialkyne substituted 2-aminobenzothiazoles. Here in, we synthesized 2-azidobenzothiazole derivatives which can react with different mono-propargylated and bis-propargylated compounds. The immense importance of 2-azidobenzothiazoles will further proceed when we apply them to the synthesis of pharmacologically significant heterocyclic scaffolds. This may provide an extra credential for the development of innovative protocols which might be worthy to cure diseases of new origin.

## 2 Materials and methods

### 2.1 Chemistry

Chemicals and solvents used in this study were purchased from E. Merck (India) and Sigma Aldrich chemicals and used without further purification. Precoated aluminum sheets (Silica gel 60 F254, Merck Germany) were employed for thin-layer chromatography (TLC). The synthesized compounds were visualized on TLC using ultraviolet (UV) light (*λ* = 254 nm). The melting points of all the compounds were observed on a Veego instrument with model specifications REC-22038 A2 and are uncorrected. ^1^H NMR was recorded on Bruker Advance DX spectrometer at 400 MHz, respectively with chemical shifts reported relative to residual deuterated solvent peaks. Splitting patterns are indicated by s (singlet), d (doublet), t (triplet), q (quartet), dd (doublet of doublet), or m (multiplet). Mass spectra were recorded by ESI-MS (AB-Sciex 2000; Applied Biosystem) and on a GCMS-QP 5000 (Shimadzu) mass spectrometers; Infra-red spectra were recorded on Perkin Elmer FT-IR spectrometer in the range of 4,000–600 cm^−1^ as neat samples.

### 2.2 *In Vitro* antibacterial activity

#### 2.2.1 Chemicals and reagents

Mueller Hinton Broth (MHB), Nutrient Agar (NA), Gram Staining Kit, and Crystal Violet (cell culture tested) used in the study were supplied by HiMedia. Dimethyl sulfoxide (DMSO), Methanol, Glycerol [Aqueous] acetic acid, Sodium Chloride (NaCl), Isopropanol, Absolute Ethanol, Resazurin, and 3-(4,5-dimethylthiazol-2yl)-2,5-diphenyl tetrazolium bromide (MTT) was purchased from Merck (India). 4′,6-diamidino-2-phenylindole (DAPI) Stain and Propidium Iodide counter stain (PI) were purchased from Thermo Fischer Scientific. Foetal Bovine Serum (FBS), Bovine Serum Albumin (BSA), Dulbecco’s Modified Eagle Medium (DMEM), Trypsin, phosphate-buffered saline (PBS) were obtained from Gibco (Thermo Fischer Scientific). All chemicals used were of analytical research grade.

#### 2.2.2 Bacterial strains

Gram (+) bacteria; *Staphylococcus aureus* (ATCC 25923), *Enterococcus faecalis* (ATCC 51299), *Bacillus cereus* (MTCC 121) and Gram (−) bacteria; *Escherichia coli* (ATCC 10536), *Pseudomonas aeruginosa* (ATCC 10145), *Klebsiella pneumonia* (NCTC 13440) were frozen as glycerol stocks and stored at −80°C. For each experiment, the frozen bacterial stock had been thawed and grown in MHB to the mid-logarithmic phase of growth under proper incubated conditions and then used.

#### 2.2.3 Determination of minimum inhibitory concentration (MIC)

To determine the MIC, the broth micro-dilution method was used, as described previously (Ali et al., 2022). The 96-well plates were labeled with the compound name, reference antibiotics (two wells/compound), and name of bacterial strains. 200 µL of MHB were dispensed into the wells except for the first column. 200 µL of the respective compounds (**2a-2h**) and drugs (CIP, AMK, and STR) were pipetted into the first column with a concentration equal to 128 μg/mL 2-fold serial dilution was performed so that the concentration from the first to 10th well ranges from 128–0.25 μg/mL. This was followed by inoculation of 50 µL of 10⁵ CFU/mL cells of *S. aureus*, *E. faecalis*, *B. cereus, E. coli*, *P. aeruginosa,* and *K. pneumonia* to wells in individual 96-well plates. The last two wells were taken as Growth Control (GC) and Media Control (MC), one lacking the drug and the other lacking both drug and compound as an indicator of contamination. The plates were sealed with parafilm. After 24 h of incubation at 37°C, plates were checked for the presence or absence of visible growth by comparing them with the Culture Control (CC) and MC Wells which also act as indictors of contamination. MIC was defined as the lowest concentration of compound at which no bacterial growth was visualized.

##### 2.2.3.1 Spectrophotometric analysis

The Optical Density (OD) of each well was measured at a wavelength of 600 nm using a microplate reader. The OD values for each well were recorded and the percentage of growth inhibition was calculated as described. In the calculation of percentage inhibition, the control well containing the bacterial culture but without any compound (GC) was used as the reference for comparison. This control well was used to determine the baseline OD value of the bacterial culture and considered as 100% growth or metabolic activity. The OD value of each well was then compared to the OD value of the control well to calculate the percentage inhibition of bacterial growth or metabolic activity. The percentage of growth inhibition in each well was calculated by comparing the OD of each well to that of the growth control wells using the following formula:

$$Percentage\;inhibition=\left[\left(OD\;control\;–\;OD\;treated\right)/OD\;control\right]x\;100$$ (1)

The data was plotted to determine the percentage of inhibition (plotting the percentage of inhibition *versus* the concentration of the antibiotic). The IC_50_ value was determined from the curve as the concentration of the antibiotic that inhibits 50% of bacterial growth.

#### 2.2.4 Determination of minimum bactericidal concentration (MBC)

To ascertain if the compounds are bactericidal, the MBC assay was performed as per CSLI, 2016 guidelines (Mir et al., 2023). MBC is the lowest concentration of compound that leads to a 3 log10-fold decrease in CFU units or the lowest drug concentration that results in 99.9% killing of bacterial cells in initial inoculums. It is important to determine MBC as it provides insights into the optimal dosing regimen and identifies potential resistance mechanisms. The MBC was performed by broth micro dilution. The experiment was performed in duplicates. Briefly, 2 sterile 96-well plates were filled with MHB containing serial two-fold dilutions ranging from 1x- 4x MIC of compounds (**2a-2h**) and Ampicillin (4–16 μg/mL) as standard in a set of 2. Drug-free controls were also included. The plates were inoculated with 50 µL of *S. aureus* and *E. faecalis* respectively with a final density equal to 10^5^ CFU. The initial CFU of inoculums was verified by plating onto NA media. After 24 h, the drug-containing wells showing no sign of growth compared to growth culture tubes were appropriately diluted to sub-MIC levels by 10-fold serial dilution in MHB. 100 μL of each dilution was plated onto NA plates in duplicates. CFU was enumerated after 24 h of incubation at 37°C. CFU/mL was calculated using the formula:

No. of Colonies×Dilution Factor÷Volume of Culture Plated (2)

Then log_10_ values of an average number of colonies and log reduction were calculated using log calculator Omni Calculator to determine if there is a 3-log reduction in cell number. Ampicillin (AMP) was used as a control drug. Colonies ranging from 30–300 were considered countable. The lowest concentration of the drug which caused 99.9% killing with a 3-log reduction in CFU was taken as MBC of the drug.

#### 2.2.5 Time kill kinetics

To ascertain the bactericidal mechanism, *i.e.*, the Time v/s Concentration dependent-killing of the lead compound **2d**, the kill curve kinetics assay, was performed using the CFU/mL method as per CSLI guidelines with slight modifications. Another method adapted from the protocol described by Wang was also used to study the kill curve to provide insights into the degree of killing (concentration dependence) and rate of killing (time dependence) (Tsuji et al., 2008).

##### 2.2.5.1 Assessment of 24-hour time-kill kinetics via CFU/mL evaluation

To determine the kill kinetics of lead **2d** compound, *E. faecalis* and *S. aureus* cultures with cell density 1 × 10⁵ CFU/mL (confirmed by plate count) were exposed to 8 μg/mL MIC, 16 μg/mL (2xMIC) and 32 μg/mL (4xMIC) of **2d**. A growth control CC was included as negative control (devoid of the compound). 2xMIC of AMP was taken as a standard drug. The log CFU/mL for all groups was determined at time 0 and at a subsequent time interval of 4 h (taken up to 24 h). Aliquots (100 µL) of drug-exposed cultures were pipetted out at predetermined time intervals of 4 h in 1 day, i.e., at 0, 4, 8, 9, 12, 16, 20, and 24 h and serially diluted, then plated onto NA for CFU enumeration. First, the colonies at different dilutions for each concentration were enumerated then the average colony count at dilutions 10^–1^, 10^–2,^ 10^–3^, and 10^–4^ was calculated and then converting the value into log_10_ (Note: the colonies on plates showing 30–300 colonies were only considered). The curve was generated by plotting Log_10_ CFU values at each concentration against time. Time-kill curves were plotted and analyzed for the rate and extent of bacterial killing. The rate of killing was determined from the start of 0 h to maximal reduction in log_10_ CFU/mL.

##### 2.2.5.2 Evaluation of time-kill kinetics based on MTT-reduction on a 360-min scale

For this assay, working solutions of **2d** were prepared in a series of conical tubes containing MIC, 2xMIC, and 4xMIC in 5 mL MHB. For each concentration, a set of tubes labeled with the time point were taken. 100 µL of 10 CFU/mL of *E. faecalis* and *S. aureus* were dispensed in their respective set of tubes and incubated at 37°C. At the various time points 0, 60, 120, 180, 240, 300, and 360 min, the incubated tubes containing culture-molecule mixtures were treated with 150 µL of MTT and incubated for 20 min. After incubation, the tubes were centrifuged at 10,000 rpm at 10°C for 10 min. The supernatant was discarded and the pellet containing formazan crystals at the bottom was dissolved in 1.5 mL of Isopropanol and absorption was measured at 550 nm. For positive control, the cells were treated with 2xMIC of STR. The untreated cultures were taken as a negative control. MTT can be reduced to purple formazan crystals by a dehydrogenase system of active cells which can be quantified through spectrophotometric analysis by dissolving in organic solvent Isopropanol. OD550 nm can be directly correlated to the number of metabolically active cells and this was used to determine the bacterial killing at various concentrations over time.

#### 2.2.6 Post-antibiotic effect (PAE)

The PAE was determined as per the described protocol (Proma et al., 2020). Bacterial samples can have persistent subpopulations that remain dormant under antibiotic treatment and lead to disease recurrence following cessation of antibiotic therapy. The PAE was determined by the viable count method and the procedure adopted was the slight modification of protocol as reported previously. Briefly, an exponentially grown culture of *E. faecalis* and *S. aureus* with a final density of 10⁶ CFU/mL were exposed to **2d** and control drug Ampicillin (AMP) at x, 2x, and 4xMIC for 2 h. After drug exposure, the drugs were removed by three cycles of centrifugation at 10,000 rpm for 10 min. The pellet obtained was resuspended in MHB. Antibiotics were removed immediately after drug exposure (2 h) and taken as 0-h readings and then after 1, 2, 3, 4, 5, 6, 7, 8, 12, and 24 h by centrifugation. Aliquots removed at predetermined time points were serially diluted 10-fold (N, 10^–1^, 10^–2^, 10^–3^, 10^–4^, 10^–5^, and 10^–6^) and plated on NA media in triplicates. Growth Controls with inoculums but no antibiotics were similarly processed and included to monitor the killing effect due to antibiotics to avoid any error in CFUs during processing.

The PAE was defined following Craig and Gudmundsson as

PAE=T−C (3)

where T is the time required for the viability count of an antibiotic-exposed culture to increase by 1 log10 above the count observed immediately after dilution and C is the corresponding time for the growth control.

#### 2.2.7 Fluorescent microscopy

DAPI and PI staining is commonly used to assess cell viability and the effect of antibiotics on cells. DAPI (4′,6-diamidino-2-phenylindole) is a fluorescent dye that binds to DNA and emits blue fluorescence upon excitation by ultraviolet light. PI (propidium iodide) is also a fluorescent dye that binds to DNA, but it emits red fluorescence upon excitation by ultraviolet light. In live cells, the DAPI stain enters the nucleus and binds to DNA, whereas PI is excluded from the cell. However, in dead or damaged cells, the cell membrane becomes permeable to PI, which enters the cell and binds to DNA, causing it to emit red fluorescence. When using DAPI and PI staining to assess cell viability and the effect of antibiotics on cells, the cells are typically treated with the antibiotic and then stained with DAPI and PI. Live cells will appear blue, whereas dead cells will appear red. The staining was done as per the described protocol (Zotta et al., 2012). This staining method was used to study the effect of lead compound **2d** on the viability of *E. faecalis* and *S. aureus* using DAPI and PI stains. Bacterial cells were grown to an OD600 of 0.5. 1 mL of the actively growing cultures was pipetted into Eppendorf tubes with MIC and 2xMIC of compound **2d**. After incubation of 2 h at 37°C, the cultures in tubes containing **2d** were centrifuged at 10,000 rpm for 10 min supernatant was decanted and the pellet was resuspended in 200 µL Normal Saline Solution (NSS). 20 µL of PI (5 μg/mL) were added to the tubes and incubated in the dark at 0°C for 15 min. The unbound PI was removed by centrifuging the tubes at 10,000 rpm for 10 min and washing the pellet thrice with 200 µL of NSS. This was followed by 15-min incubation with 2 µL of DAPI (10 μg/mL) at 0°C in the dark. Finally, 100 µL of cultures were mounted onto the clean well-labeled slides, left to dry, and methanol fixed. For negative controls, the corresponding bacterial cells that did not receive **2d** treatment were processed under similar conditions. The stained bacterial cells were visualized under a fluorescent microscope and photographed using an inbuilt digital camera.

#### 2.2.8 Microtiter plate biofilm inhibition assay

To determine whether compound **2d** can inhibit the formation of biofilms, the Microtiter Plate Biofilm Assay was utilized as per described procedure (O’Toole, 2011; Mir et al., 2023). Firstly, the compound was prepared as a working stock (200 µL of compound in first well) and was serially diluted two-fold to obtain a concentration ranging from 0.25 to 128 μg/mL in 8 individual 96-well plates (1 plate for inoculation of each bacterium) already containing 100 µL MHB (final volume of 200 µL). 50 µL of bacterial suspension containing 10⁵ CFU/mL bacteria cells were added to the wells of their respective plates (*S. aureus, E. faecalis, B. cereus, E. coli, P. aeruginosa, K. pneumonia*). Culture control and Media control were included, in which only bacterial culture and growth media, respectively, were dispensed. The plates were then incubated at 37°C for 24 h to allow bacterial growth and understand if biofilm formation was inhibited in the presence of the compounds, as specified. No visible growth was observed in wells that correspond to the minimum inhibitory concentration (MIC) of the compound for the bacterial strains tested. After the incubation period, the planktonic bacterial cells in the wells were removed by washing three times with phosphate-buffered saline (PBS), and aliquots from the wells were serially diluted and plated onto NA to correlate the further findings. Next, 125 µL of 30% of 0.5% crystal violet (CV) that included methanol was added to all the wells of the 96-well plates. The plate was then incubated at 37°C for 20 min to stain the biofilm formed by microorganisms that are adherent to the abiotic surface of a microtiter plate. The unbound CV was removed by washing the wells with distilled water, and the wells were air-dried at room temperature. The wells were then visualized and photographed under a microscope to determine the extent of biofilm formation. To quantify the amount of biofilm formed, 200 µL of 30% [aqueous] acetic acid was added to each well to dissolve the crystal violet. The optical density (OD) of the dissolved CV was measured at 470 nm using a microplate reader. This protocol was repeated thrice to achieve optimal results and correct any background interference that may be present. The percentage of inhibition of biofilm formation was calculated using the following formula:

$$\left[100-\left({\text{TreatedOD}}_{470}\text{nm}÷\text{ Growth }{\text{ControlOD}}_{470}\text{nm}\right)\;\times 100\right]$$ (4)

This formula compares the optical density of the treated wells to that of the culture control wells and expresses the result as a percentage of inhibition.

#### 2.2.9 Biofilm eradication assay

A modified methodology incorporating diverse methods used earlier was implemented to determine the potential of compound **2d** for biofilm eradication and assess the minimum concentration required for biofilm eradication (Kreth et al., 2005). 12 well plates were employed for this assay. To begin with, the bacterial strains (*S.aureus*, *E. faecalis*, *B. cereus*, *P. aeruginosa*, *E. coli*, and *K. pneumonia*) were grown to mid-log phase and diluted to obtain 10⁵ CFU/mL of cells. 3 mL of MHB was pipetted into the 12 well plates and subsequently inoculated with 100 µL of the bacterial suspension. Media Control well was taken as a positive control containing only MHB. For each bacterial strain, separate plates were utilized and subjected to incubation for 24, 48, and 72 h to assess the effect of different concentrations on various stages of bacterial biofilm formation. After the incubation at given time points the plates were washed thrice with PBS to remove the planktonic bacterial cells. After washing x, 2x, 4x, 8x, and 16x MIC concentrations of **2d** compound were added to the wells and incubated for 24 h at 37°C. Culture Control well taken as a negative control was not incubated with the compound. After incubation plates the drug-containing plates were washed with PBS. This was followed by the addition of 30% of 0.5% CV stain. The plates were further incubated for 1 h at room temperature. In the next step, the CV was removed by thorough washing. Each well was observed under a microscope and photographed. To quantify the percentage of eradication 30% [aqueous] acetic acid was added to the wells to dissolve CV-staining adherent cells. This was done by recording the OD at 470 nm using a spectrophotometer. The percentage of biofilm eradication was calculated using formula **(4)**. The percentages were calculated by taking the mean of three OD readings obtained for each concentration.

#### 2.2.10 Cytotoxicity assay

The critical factor that allows any compound to proceed successfully during drug discovery is selective toxicity to pathogens and non-toxicity to human cells. The assay was performed under the standards set by International Organization for Standardization (ISO) and Economic Cooperation and Development (OECD), to ensure accurate and reproducible results. The cytotoxicity of the lead compound **2d** was evaluated against HEK-293. Before evaluating the cytotoxicity of the compound, the cells were grown to reach a confluency of 70%–80%. The culture techniques used were in accordance with the cell culture technique guidelines regulated by the International Cell Line Authentication Committee (ICLAC), American Type Culture Collection (ATCC), and the European Collection of Authenticated Cell Cultures (ECACC).

##### 2.2.10.1 Cell lines

Human Embryonic Kidney Cell lines, HEK-293 were purchased from National Centre for Cell Sciences (NCCS), Pune, India. The cell lines were cultured in DMEM (supplemented with 10% foetal bovine serum) in a Carbon Dioxide incubator with 98% humidity and 5% CO_2_ at 37°C.

##### 2.2.10.2 Preparation of HEK-293 for cytotoxicity evaluation

Thawing of frozen cells: HEK-293 cells stored at −80°C were thawed by removing the frozen cryovial and thawing it in 37°C water for 1–2 min until no visible was seen in the vial. The vials were swirled gently to ensure that the cells are fully thawed. Once the cells were thawed, the vial was removed from the water bath and wiped with 70% ethanol to sterilize the outside of the vial.

Cell Culture and Expansion: The thawed HEK-293 cells were then transferred to a sterile hood and the contents of the vial were transferred into a sterile tube containing 9 mL of pre-warmed growth medium Dulbecco’s Modified Eagle’s Medium (DMEM) supplemented with 10% FBS, and 1% penicillin-streptomycin. This was followed by centrifuging the tube at 1,000 rpm for 5 min to pellet the cells. The supernatant was aspirated and the cell pellet and resuspended in 2 mL of fresh pre-warmed growth medium. The cell suspension was transferred to a T75 culture flask and incubated at 37°C with 5% CO_2_ until they reached the desired confluency of 70%–80%.

Harvesting of cells: Once the cells had reached the desired confluency, they were harvested using standard trypsinization techniques. A small volume of Trypsin-EDTA solution was added to the culture flask and incubated at 37°C for 2 min. During incubation, the Trypsin cleaves the proteins that attach the cells to the culture surface, causing the cells to detach from the surface. The culture medium was removed from the culture dish, and the cells were washed with phosphate-buffered saline (PBS) to remove any residual serum. After incubation, an equal volume of culture medium containing FBS and DMEM was added to neutralize the Trypsin and stop the enzymatic activity. The cells were then collected by gently tapping or swirling the culture dish and transferred to a centrifuge tube. The cells were again pelleted by centrifugation, and the supernatant is discarded. The cells were resuspended in a fresh culture medium, counted, and seeded into a new culture flask. The harvested cells were then counted using a hemocytometer. A predetermined number of cells were seeded into 96-well optimized for the cytotoxicity assay being performed.

##### 2.2.10.3 Cytotoxicity assay using MTT

The method was carried out as described earlier (Adan et al., 2016). The 100 µL of HEK-293 cells were seeded into the 96-well microtiter plate and allowed to grow for 24 h at 37°C with 5% CO_2_. The cells were treated with 6 concentrations of the **2d** compound 4, 8, 16, 32, 64, and 128 μg/mL dissolved in DMSO for time points 24, 48, and 72 h to determine the time and dose dependent effect of the compound on cell viability. Untreated control wells received only DMSO. After the given time points, the number of viable cells was determined. Briefly, the tissue culture medium was removed from the 96-well plate and replaced with 100 µL of fresh medium then 20 µL of the MTT stock solution (5 mg of MTT in 1 mL of PBS) was added to each well including the untreated controls. The 96-well plates were then incubated at 37°C and 5% CO_2_ for 4 h. An aliquot (85 µL) of the medium was removed from the wells, and 150 µL of dimethyl sulfoxide was added to each well to dissolve the insoluble formazan crystals formed by viable cells and mixed thoroughly with the pipette and incubated at 37°C for 10 min. The optical density was measured at 590 nm with a microplate reader to determine the number of viable cells based on the selective ability of viable cells to reduce the tetrazolium component of MTT into purple-colored formazan crystals. The colored formazan intensity was measured at 570 nm absorbance in the micro-plate reader and growth percentage inhibition was calculated using the formula:

$$Mean\;{OD}_{570}\;Treated\;Well÷Mean\;{OD}_{570}\;Control\;well\times 100$$ (5)

The results expressed were the average values of three experiments (±SD). Reduction of MTT to formazan crystals is mediated by the mitochondrial enzyme succinate dehydrogenase of cells the viable cells. The color of the formazan crystals that form after the reduction of MTT by viable cells indicates the level of cell viability and, hence, the cytotoxicity of the test compound. The formazan crystals appear as purple or dark blue precipitates in viable cells. The intensity of the color is directly proportional to the number of viable cells in the culture. Conversely, a decrease in the intensity of the color indicates a decrease in the number of viable cells and, hence, an increase in cytotoxicity. A complete absence of color indicates that all the cells in the culture have died, and the test compound is highly cytotoxic to the cells.

#### 2.2.11 General procedure for the synthesis of 2-azidobenzothiazoles

2-aminobenzothiazole (0.5 mmol) was dissolved in water (10 mL) in a 50 mL round bottom flask under a magnetic stirrer at room temperature; to this solution, hydrochloric acid was added dropwise until 2-aminobenzothiazole gets completely dissolved in water. In the next step solutions of sodium nitrite, (2 mmol), sodium acetate (2 mmol), and sodium azide (2 mmol) were added dropwise after every 5 min respectively and the reaction was stirred for further 30 min. The reaction progress was checked by thin layer chromatography which confirmed the completion of the reaction within 1 h. The solid precipitate was filtered, washed with water, and recrystallized from ethanol which did not require any further purification. (Note: *NaNO*_*2*_ *, NaOAc, and NaN*_*3*_ *were first dissolved in water, and then added dropwise to the reaction mixture respectively. Also, the role of sodium acetate is to neutralize the acidic medium*).

#### 2.2.12 Spectroscopic data

##### 2.2.12.1 2-Azido-4, 6-difluorobenzothiazole (2a)

Brown solid, yield: 93%; mp 170°C–173°C; IR (neat) ʋ: 2,124 cm^−1^. ^1^H NMR (400 MHz, DMSO-*d*_*6*_) δ (ppm): 7.98 (m, 1H), 7.63 (m, 1H). MS *(m/z)* 234.88 (M.F.:C_7_H_2_F_2_N_4_S).

##### 2.2.12.2.2-Azido-6-methoxybenzothiazole (2b)

Brown solid, yield: 93%; mp 155°C–158°C; IR (neat) ʋ: 2,113 cm^−1^. ^1^H NMR (400 MHz, DMSO-*d*_*6*_) δ (ppm): 8.25 (d, 1H), 7.94 (d, 1H), 7.35 (dd, 1H), 3.93 (s, 3H). MS *(m/z)* 207.03 (M.F.: C_8_H_6_N_4_OS).

##### 2.2.12.3 Ethyl 2-azidobenzothiazole-6-carboxylate (2c)

White solid, yield: 89%; mp 145°C–148°C; IR (neat) ʋ: 2,126 cm^−1^. ^1^H NMR (400 MHz, CDCl_3_) δ (ppm): 8.08 (d, 1H), 7.47 (dd, 1H), 7.39 (d, 1H), 3.74 (q, 2H), 1.28 (t, 3H).

##### 2.2.12.4 2-Azido-6-nitro-benzothiaole (2d)

Creamy white solid, yield: 95%; mp 190°C–193°C; IR (neat) ʋ: 2,120 cm^−1^. ^1^H NMR (400 MHz, CDCl_3_) δ (ppm): 8.77 (d, 1H), 8.40 (dd, 1H), 8.10 (d, 1H). MS *(m/z)* 222.03 (M.F.:C_7_H_3_N_5_O_2_S).

##### 2.2.12.5 2-Azidobenzothiazole (2e)

Brown solid, yield: 80%; mp 153°C–156°C; IR (neat) ʋ: 2,119 cm^−1^. ^1^H NMR (400 MHz, CDCl_3_) δ (ppm): 8.22 (d, 1H), 7.74 (d, 1H), 7.70–7.58 (m, 2H). MS *(m/z)* 176 (M.F.: C_7_H_4_N_4_S).

##### 2.2.12.6 2-Azido-4-methylbenzothiazole (2f)

Orange solid; yield: 80%; mp 117°C–120°C; IR (neat) ʋ: 2,120 cm^−1^. ^1^H NMR (400 MHz, CDCl_3_) δ (ppm): 7.73 (d, 1H), 7.30 (d, 1H), 7.18 (t, 1H), 2.70 (s, 3H). MS *(m/z)* 191 (M.F.: C_8_H_6_N_4_S).

##### 2.2.12.7.2-Azido-6-ethoxybenzothiazole (2g)

Brown solid, yield: 92%; mp 161°C–164°C; IR (neat) ʋ: 2,107 cm^−1^. ^1^H NMR (400 MHz, CDCl_3_) δ (ppm): 8.07 (d, 1H), 7.70 (d, 1H), 7.02 (dd, 1H), 4.06 (q, 2H), 1.44 (t, 3H). MS *(m/z)* 221 (M.F.: C_9_H_8_N_4_OS).

##### 2.2.12.8.2-Azido-5-bromobenzothiazole (2h)

Brown solid; yield: 82%; mp 180°C–183°C; IR (neat) ʋ: 2,110 cm^−1^. ^1^H NMR (400 MHz, DMSO-*d*_*6*_) δ (ppm): 8.32 (d, 1H), 8.16 (d, 1H), 7.66 (dd, 1H).

## 3 Results and Discussion

### 3.1 Chemistry

**SCHEME 1:**
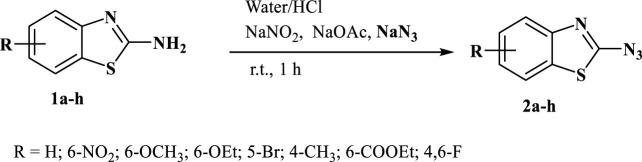
2-aminobenzothiazoles converted into 2-azidobenzothiazoles using sodium nitrite and sodium azide.

**FIGURE 1:**
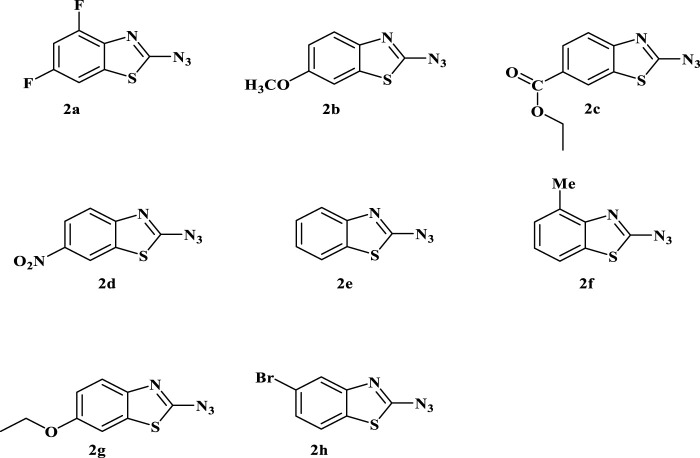
Depiction of the synthesized 2-azidobenzothiazoles. Reaction conditions: Substrate (0.5 mmol, 1equiv), NaNO_2_ (2 mmol, 4equiv), NaOAc (2 mmol, 4equiv), NaN_3_ (2 mmol, 4equiv), H_2_O/HCl, r.t., 1 h. Isolated yield and structure were confirmed by comparison of IR, mp, MS, and ^1^H NMR. (**Note:** Although we have not experienced any problem in handling this compound, precautions should be taken due to its explosive nature).

This study presents a simple approach for the synthesis of 2-azidobenzothiazoles through the transformation of substituted 2-aminobenzothiazoles. The method involves the utilization of sodium nitrite and sodium azide as key reagents under mild reaction conditions (Scheme 1). The introduction of azide groups into organic molecules, facilitated by sodium nitrite and sodium azide, is a significant chemical transformation that opens doors to diverse functionalization and subsequent applications. The mild reaction conditions employed, in conjunction with these reagents, contribute to the synthesis’s accessibility and potential applicability across a range of substrates. The synthesis of 2-azidobenzothiazoles via (Figure 1) this methodology adds to the toolbox of functional group transformations. The ability to incorporate azide functionality onto the benzothiazole scaffold is noteworthy due to the versatile reactivity of azides, which can be harnessed for further derivatization and diversification of the synthesized compounds. Additionally, the utilization of substituted 2-aminobenzothiazoles as starting materials allows for the introduction of specific substituents that can influence the resulting compound’s properties and behaviors. The mild conditions employed in this synthesis are advantageous for maintaining the integrity of sensitive functional groups and minimizing unwanted side reactions. This feature is particularly beneficial when working with complex or delicate molecules, where harsher conditions might lead to undesired chemical transformations or yield losses. Further analysis of the equal amounts of sodium nitrite, sodium acetate, and sodium azide necessary for the reaction revealed that 4 equivalents of each were suitable to achieve the desired product in excellent yield. The synthesis of 2-azidobenzothiazoles in an isolated yield of 80%–95% results from the smooth, rapid, and quantitative progression of the reaction. It is evident that both electron donating and electron withdrawing substrates are acceptable for this transformation and generated good to excellent yields of the intended product in 1 h, establishing the further scope of the reaction. It was intriguing to learn that free ester and halogen groups might withstand the conditions of a reaction without producing hydrazide.

The choice of substituents and substitution patterns in the synthesis of 2-azidobenzothiazoles, as well as the subsequent antibacterial activity studies, seems to be guided by specific factors aimed at enhancing the antibacterial potential of the compounds while minimizing cytotoxicity. The selection of certain substituents and substitution patterns has been inspired by previous studies or analogs that have shown promising antimicrobial activities. The presence of electron-withdrawing groups like -NO₂ at the *meta* position increases the antibacterial activity by influencing interactions with bacterial targets. In this context, the introduction of a nitro group (-NO₂) on the benzothiazole ring might enhance antibacterial potential. Replacement of the *meta* substituent of benzothiazole ring with methoxy group (**2b**), carboxylate (**2c**), hydrogen (**2e**), or ethoxy decreased the activity. Conversely, introducing a 4,6-difluoro group (**2a**) on the benzothiazole ring did not show any enhancement in the activity. In addition to this, the presence of the halogen group at the *ortho* position and a methyl group at the *para* position of the benzothiazole ring do not exhibit significant effects on the activity. The capability of 2-azido-6-nitro-benzothiazole to inhibit biofilm formation is noteworthy. This indicates potential interference with bacterial adhesion and colonization, an important factor in bacterial pathogenicity. The study’s focus on extended exposure of a human cell line (Human Embryonic Kidney-293) to higher concentrations of 2-azido-6-nitro-benzothiazole suggests an attempt to determine the compound’s selectivity for bacterial cells over human cells. The fact that the compound demonstrates selective cytotoxicity towards bacterial cells even at higher concentrations is promising for potential therapeutic applications.

### 3.2 *In Vitro* antibacterial activity

#### 3.2.1 Minimum inhibitory concentration (MIC)

The Antibacterial Activity and MIC of all the synthesized compounds were determined using the Broth Micro dilution method as described in the methodology. All the synthesized compounds were tested for their antibacterial activity against the American Type Culture Collection (ATTC) strains of *S. aureus, E. faecalis, B. cereus, K. pneumonia, P. aeruginosa* and *E. coli* and clinical isolates of Methicillin Resistant *S. aureus* and Multidrug Resistant *E. coli*. It was found that compound **2d** showed good antibacterial activity against the Gram (+) bacterial strains. It was elucidated that the compounds are more active against the Gram (+) bacterial strains, *E. faecalis,* and *S. aureus* with MIC of 8 μg/mL (Table 1). However, it was moderately active against Gram (−) bacteria *P. aeruginosa* and Gram (+) bacteria *B. cereus* with a MIC value of 64 μg/mL. Substantially the compound showed activity even against the Resistant strains, MRSA, and MDR *E. coli* of clinical origin with a MIC value of 128 μg/mL (Tables 1, 2). These results were found to be consistent with several other benzothiazole derivatives, which inhibited the growth of Gram (+) and Gram (−) bacteria (Catalano et al., 2013; Singh et al., 2013). The MIC values of control drugs of known MIC values against the bacterial strains were also similar to the range given by CSLI (Clinical And Laboratory Standards Institute).

**TABLE 1:** MIC (µg/mL) of the compounds and antibiotics tested against Gram (+) bacterial strains. Amikacin (AMK), Streptomycin (STR) and Ciprofloxacin (CIP) were used as controls.

S. No.	Compound	*S. aureus*	*E. faecalis*	*B. cereus*	*MRSA*
1	**2a**	128	64	128	128
2	**2b**	128	128	128	128
3	**2c**	64	64	128	128
4	**2d**	**8**	**8**	64	128
5	**2e**	128	32	64	128
6	**2f**	128	>128	128	128
7	**2g**	128	128	128	128
8	**2h**	128	128	128	128
9	AMK	2.5	2.5	1.25	>128
10	STR	2.5	2.5	2.5	>128
11	CIP	1.25	0.3125	2.5	>128

The bold values in the tables depict MIC, MBC in low range.

**TABLE 2:** MIC (µg/mL) of the compounds and antibiotics tested against Gram (−) bacterial strains. Amikacin (AMK), Streptomycin (STR) and Ciprofloxacin (CIP) were used as controls.

S. No.	Compound	*P. aeruginosa*	*K. pneumonia*	*E. coli*	*MDR E. Coli*
1	**2a**	128	128	128	128
2	**2b**	128	64	64	128
3	**2c**	128	128	64	128
4	**2d**	64	128	128	128
5	**2e**	64	128	128	128
6	**2f**	128	128	128	128
7	**2g**	128	128	128	128
8	**2h**	128	128	128	128
9	AMK	2.5	2.5	2.5	>128
10	STR	2.5	2.5	2.5	>128
11	CIP	5	1.25	0.015	>128

The bold values in the tables depict MIC, MBC in low range.

#### 3.2.2 Minimum bactericidal concentration (MBC)

As MIC only measures the ability of antimicrobial agents to inhibit bacterial growth, we performed an MBC assay which determines the lowest concentration of antimicrobial compound that can kill 99.9% of bacterial cells. MBC is required by regulatory agencies for approval of new antimicrobials. The MBC of the series of compounds **2a-h** was assessed against the Gram (+) bacterial strains *E. faecalis and S. aureus* as the compounds exhibited comparatively better MIC values against these strains compared to other strains. The MBC values must not be more than four times the MIC, which is consistent with our results (Gonzalez et al., 2013). For both *S. aureus* and *E. faecalis* MBC of the lead compound **2d**, was equal to 2xMIC, i.e., 16 μg/mL. The other compounds tested had bactericidal effects (led to 99.9% killing) rather than bacteriostatic effects, with MBC values of 128 μg/mL. Compound **2a** showed bactericidal activity at 2xMIC (128 μg/mL) against *E. faecalis*. Compound **2b** exhibited bactericidal activity at its MIC values, indicating their potential as promising antimicrobial agents that can effectively inhibit and kill bacterial pathogens. **2c** showed bactericidal potential against both Gram (+) bacteria at 2xMIC.**2e** showed bactericidal potential only against *E. faecalis* at 4x MIC. Ampicillin was used as a control drug and its MIC and MBC values against *S. aureus* were found to be 4 and 8 μg/mL and for *E. faecalis* the MIC and MBC values were 2 and 4 μg/mL. Negative controls for both strains, devoid of compounds were used to validate the CFU counts. CFU enumeration indicated a 3-log reduction, 99.9% killing in both Gram (+) bacteria compared to the CFU count of Growth control which is indicative of the MBC. Overall, these findings suggest that the tested compounds have potent bactericidal activity against *E. faecalis* and *S. aureus*, which could be crucial in eradicating bacterial infections. The MBC values obtained after the enumeration of the CFU/mL are given in Table 3.

**TABLE 3:** MBC (µg/mL) of 2-azidobenzothiazoles against *Staphylococcus aureus* and *E. faecalis.*

S. No.	Compound name	*E. faecalis*	*S. aureus*
1	**2a**	128	128
2	**2b**	128	128
3	**2c**	128	128
4	**2d**	**16**	**16**
5	**2e**	128	>128
6	**2f**	128	128
7	**2g**	128	128
8	**2h**	128	128
9	**Ampicillin**	**4**	**8**

The bold values in the tables depict MIC, MBC in low range.

#### 3.2.3 Time kill kinetics

To study the dynamics of *in vitro* activity, we assessed the kill curve kinetics of our lead compound **2d** against *S. aureus* and *E. faecalis* at concentrations of MIC (8 μg/mL), 2xMIC (16 μg/mL) and 4xMIC (32 μg/mL) for 24 h at seven time points (0, 4, 8, 12, 16, 20 and 24 h of exposure) using the two methods as described in methodology.

##### 3.2.3.1 Assessment of 24-hour time-kill kinetics via CFU/mL evaluation

###### 3.2.3.1.1 Staphylococcus aureus

**FIGURE 2:**
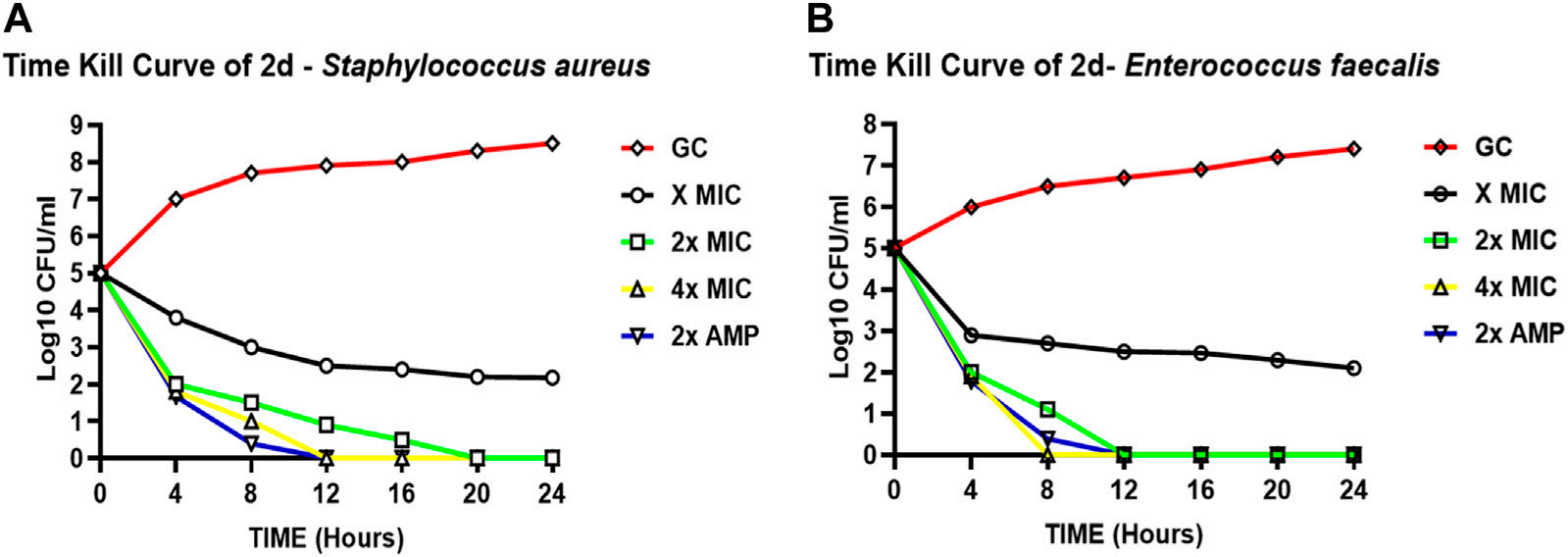
Time-kill curves of compound **2d**. The killing activity of **2d** against two bacteria against two bacterial *strains S. aureus* **(A)** and *E. faecalis* **(B)** monitored for 24 hours by CFU Enumeration method. The compound concentration used in the experiment were MIC (green), 2x MIC (pink), and 4x MIC (blue). Ampicillin indicated by the yellow line was used as a positive control. For negative control (Black line), the cultures were incubated under the similar condition without any drug.

Figure 2A represents the time kill curve of the compound **2d** against *S. aureus* at different concentrations (MIC, 2xMIC, and 4xMIC) at the given time points, compared to ampicillin (2xAMP) and growth control (GC). The colonies were counted and converted into log10 values in Excel. At time zero, all cultures showed similar bacterial counts ranging from 5.08 to 5.10 log10 CFU/mL. As time progressed, the bacterial counts decreased in all cultures except GC. The compound **2d** at 4xMIC showed the most potent activity, reducing the bacterial counts to 0.08 log10 CFU/mL after 12 h of incubation (more than 3 log reductions). In comparison, at 2xMIC, compound **2d** reduced the bacterial counts to 0.4 log10 CFU/mL within 16 h, and at MIC, the reduction was modest, with bacterial counts remaining at 2.403 log10 CFU/mL after 24 h of incubation indicating the bacteriostatic nature of the compound at a lower concentration. When comparing the activity of compound **2d** to ampicillin (2xAMP), the results showed that at 4xMIC and 2xAMP, the bacterial counts reduced to similar levels after 12 h of incubation. Moreover, at the time interval of 4–12 h maximum killing was observed at all three concentrations. In conclusion, compound **2d** showed a concentration-dependent as well as time-dependent bactericidal activity against *S. aureus*, with the highest bactericidal activity observed at 4xMIC within 12 h.

###### 3.2.3.1.2 Enterococcus faecalis

Figure 2B represents the time-kill curve of *E. faecalis*, with various concentrations of **2d**, MIC, 2xMIC, 4xMIC, and 2xAMP at different time points. The bacteria appear to be highly susceptible to the compound as evidenced by the significant decrease in the growth rate at higher concentrations over time. After an interval of 4 h, a significant reduction in the growth rate was observed at all concentrations (approximately 2-log reduction across all concentrations). After 8 h, there was a significant decrease in CFU/mL count, at higher concentrations of 2x and 4xMIC, while as for MIC the activity remained static. 4xMIC exhibited bactericidal activity achieving 99.9% killing within 8 h. At MIC the compound exhibited a bacteriostatic activity at all time points. At 2xMIC the bactericidal activity was achieved after 12 h which is similar to that of Ampicillin. The results suggest that *E. faecalis* was highly susceptible to the compound at higher concentrations and the bactericidal effect was observed at 2xMIC and 4xMIC within 12 and 8 h respectively. Overall, we can deduce from the results that the activity of compound **2d** is concentration dependent as well as time dependent killing.

##### 3.2.3.2 Evaluation of time-kill kinetics based on MTT-reduction on a 360-minute scale

**FIGURE 3:**
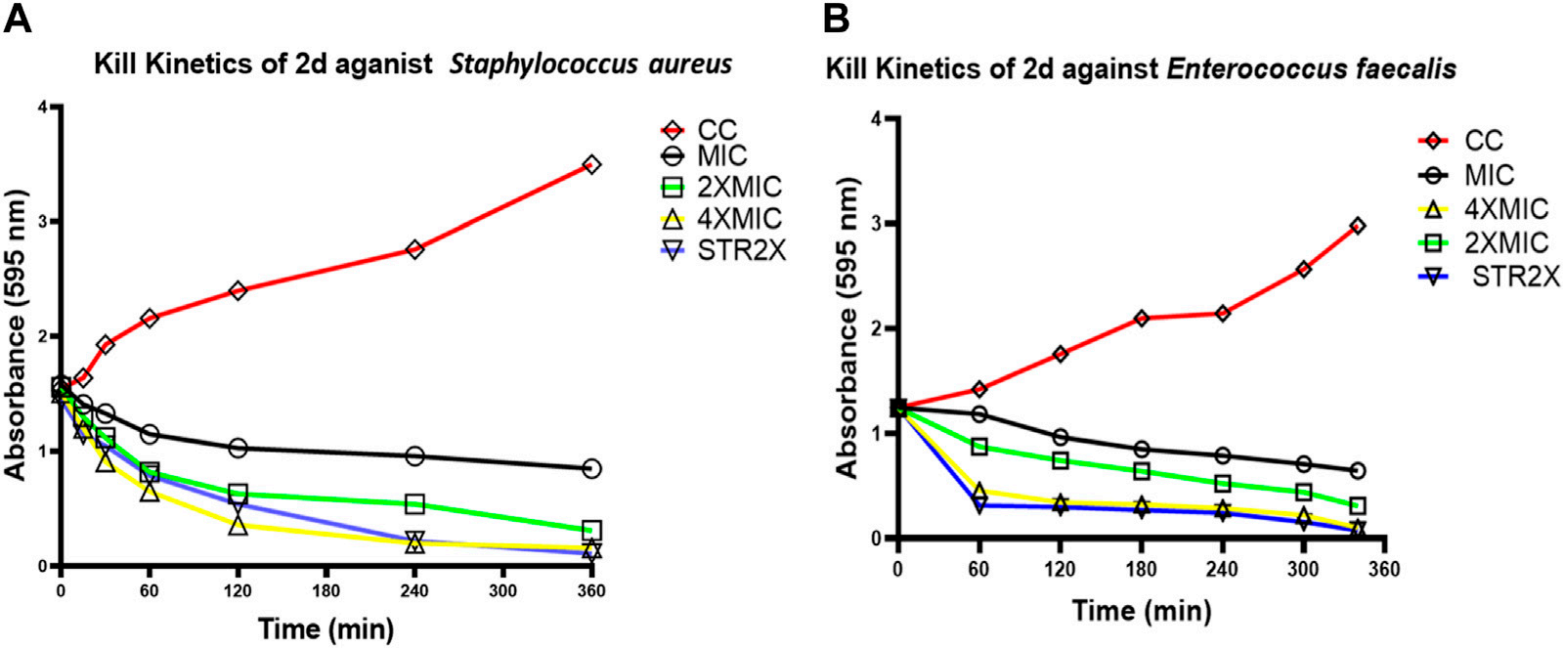
Time kill curves of **2d**. The killing activity of **2d** against two bacterial *strains S. aureus* **(A)** and *E. faecalis* **(B)** was monitored for 360 min. The compound concentration used in the experiment were MIC (Black), 2xMIC (Green), and 4xMIC (Yellow). Streptomycin (STR) indicated by the Blue line was used as a positive control. For negative control (Red line), the cultures were incubated under similar conditions without any drug.

To determine whether the effect of compound **2d** on the growth of *S. aureus* and *E. faecalis* is dose dependent, the time kill kinetic assay was carried out using MTT on the minute scale as described in the methodology. Both the bacterial strains were incubated individually with compound **2d** at its final concentration of MIC, 2xMIC, and 4xMIC. The optical density of the cultures was recorded at time intervals of 60 min. It is clear from Figure 3 that the compound showed bactericidal activity against both strains in a dose dependent manner. The killing pattern of compound **2d** against *S. aureus* was comparable to that of Streptomycin with over 75% of bacterial death within 4 h of the treatment. For *E. faecalis*, however, the compound showed a similar rate of death after 2 h of treatment compared to Streptomycin.

#### 3.2.4 Post-antibiotic effect (PAE)

To investigate the effect of compound **2d**, post its withdrawal, and assess whether the suppression in the growth of bacterial cells, *S. aureus* and *E. faecalis* persist after this brief exposure, the post-antibiotic effect (PAE) was calculated. PAE was deduced by the viable count method as described. *Staphylococcus aureus* and *E. faecalis* were individually treated with MIC (8 μg/mL), 2xMIC (16 μg/mL), and 4xMIC (32 μg/mL) concentrations of the compound **2d** and MIC of Ampicillin for 2 h. The growth was monitored by CFU enumeration after a time interval of 1 h. The PAE results represented here are determined at a higher inoculum size of 1 × 10^6^ CFU/mL as against the routine inoculum size of 1 × 10^5^ CFU/mL more commonly used for susceptibility testing (MIC). To avoid the ambiguity in PAE results owing to the higher inoculum effect, MIC assay was first performed at a higher inoculum size (1 × 10^6^ CFU/mL) to select the correct concentrations of antibacterial agents (**2d** & AMP) for PAE determination. No change in MIC of **2d** as well as Ampicillin was determined at higher inoculum size. PAE was calculated using formula (3) as described in the methodology.

##### 3.2.4.1 Staphylococcus aureus

The cells took an average of 5.33 h to increase their CFU by 1 log_10_ post-withdrawal of compound **2d**, irrespective of the concentration of compound **2d**. After treatment and removal of 8 and 32 μg/mL of **2d**, the growth was suppressed for around 6 h. The recovery period after **2d** treatment was almost identical to that of MIC of Ampicillin treatment. The PAE results indicate that compound **2d**, at concentrations ranging from 8, 16, and 32 μg/mL, demonstrates a significant and sustained inhibitory effect on bacterial regrowth even after the removal of the antibiotic. The consistent PAE duration across the tested concentrations suggests that compound **2d** exhibits a concentration-independent PAE (Table 4).

**TABLE 4:** Post antibiotic activity of 2d at MIC, 2xMIC, and 4xMIC and Ampicillin against *Staphylococcus aureus.*

Compound	MIC	Time (hours)	PAE = T-C
	1X	6	5.00
**2d**	2X	7	6.00
	4X	6	5.00
Ampicillin	2X	6	5.00

The culture controls took, **C= 1.00** h to grow in the absence of the antimicrobial agent.

The average of the PAE, of 2d at different concentrations was around **5.33 h**.

##### 3.2.4.2 Enterococcus faecalis

In the case of *E. faecalis*, the cells took an average of 5.66 h to increase their CFU by 1 log_10_ post-withdrawal of compound **2d**, which was comparable to that of Ampicillin. In this case, also the results indicate that the post-compound effect is independent of drug concentration as there is not much variation in time taken by the bacteria to increase their CFU by l log_10_ at all three concentrations tested (Table 5).

**TABLE 5:** Post antibiotic activity of 2d at MIC, 2xMIC, and 4xMIC and Ampicillin against *E. faecalis.*

Compound	MIC	Time (hours)	PAE = T-C
	1X	7	5.00
**2d**	2X	8	6.00
	4X	8	6.00
Ampicillin	2X	8	6.00

The culture controls took, **C= 2** h to grow in the absence of the antimicrobial agent.

The average of the PAE, of **2d** at different concentrations was around **5.66 h**.

**FIGURE 4:**
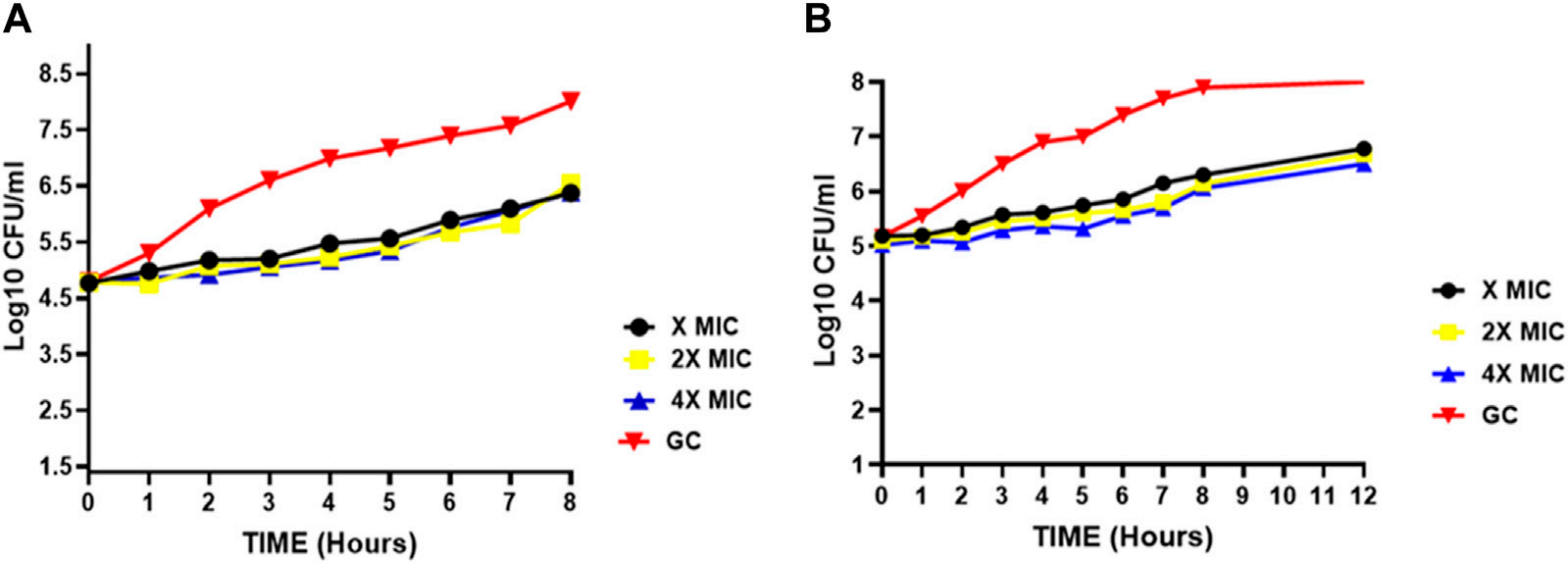
Post-antibiotic effect of compound **2d** at various concentrations against **(A)** *Staphylococcus aureus* **(B)** *E. faecalis* indicating the time points at which a 1 log10 CFU/mL increase was observed for *Staphylococcus aureus* and *E. faecalis* after a brief exposure (2 h) to compound **2d** at different concentrations (x, 2x, and 4xMIC) and subsequent removal of the antimicrobial agent.

The results reveal that both *S. aureus* and *E. faecalis* exhibited similar post-treatment growth characteristics in response to compound **2d**. The average time required for a 1 log10 CFU increase was 5.33 h (±0.577) for *S. aureus* and 5.66 h (±0.617) for *E. faecalis* when treated with MIC, 2xMIC, and 4xMIC concentrations of compound **2d** (Figure 4). These results suggest a consistent and predictable post-treatment effect independent of the drug concentration, indicating the potential effectiveness of compound **2d**. The sustained inhibitory effect of concentration-independent PAE can reduce the selection pressure for antibiotic-resistant bacterial strains. By maintaining a prolonged suppression of bacterial growth even after sub-MIC levels, the development and spread of resistance mechanisms may be hindered, preserving the effectiveness of the antibiotic over an extended period. Concentration-independent PAE offers advantages in terms of flexibility in dosing, extended suppression of bacterial regrowth, reduced resistance development, and optimized antibiotic therapy. These benefits contribute to the potential improvement in treatment outcomes and the management of bacterial infections.

#### 3.2.5 Fluorescent microscopy

**FIGURE 5:**
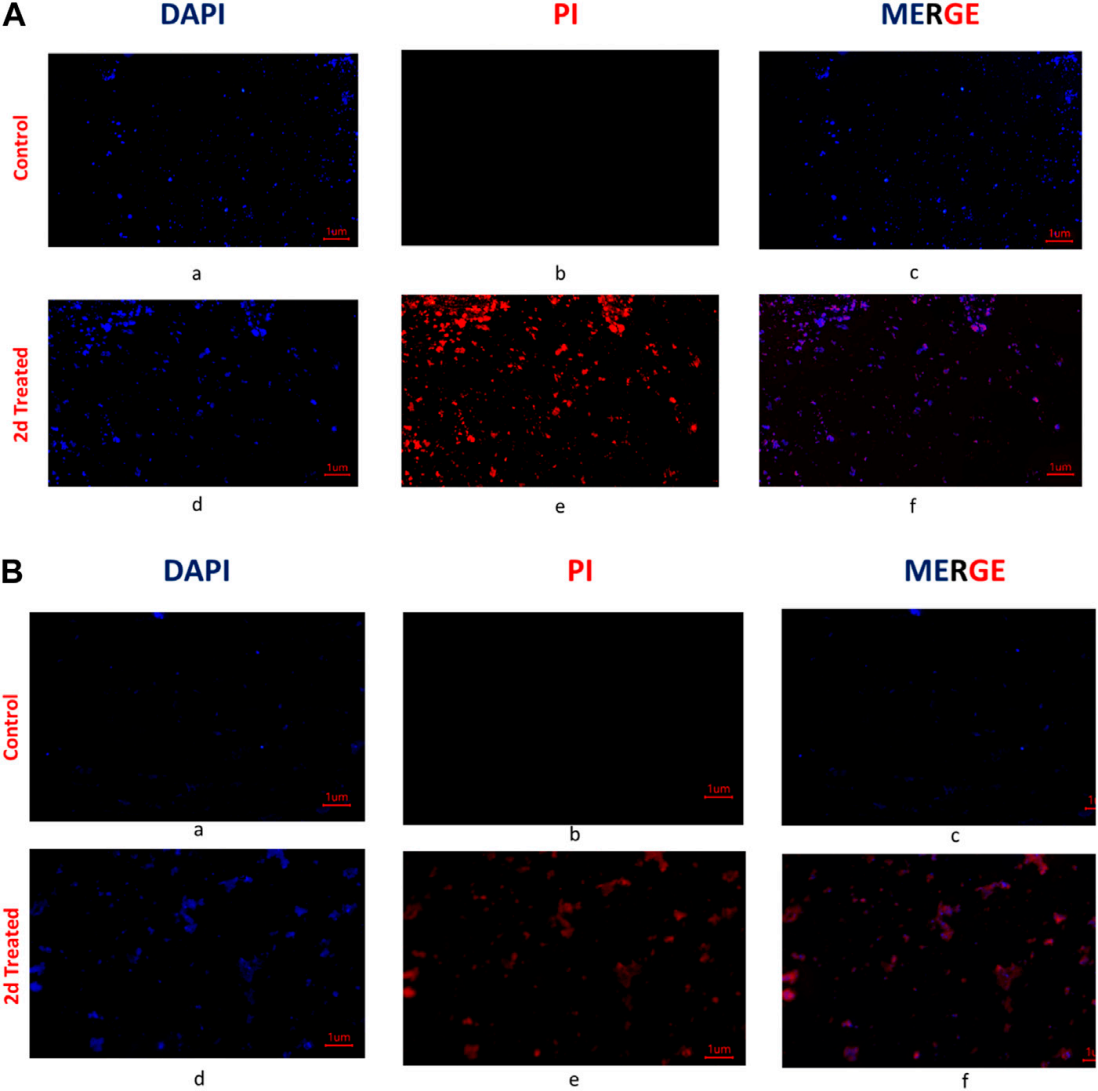
**(A)** Fluorescent images of *Staphylococcus aureus*: The untreated cells were stained with DAPI and PI and visualized under a fluorescent microscope. The photographed images of cells for DAPI (a) and PI (b) were merged (c). Under similar conditions **2d** (2x) treated *Staphylococcus aureus cells* were photographed for DAPI (d) and PI (e) and the images were merged using Image (f); **(B)** Fluorescent microscopy images of *E. faecalis:* The untreated cells were stained with DAPI and PI and visualized under a fluorescent microscope (Magnus). The photographed images of cells for DAPI (a) and PI (b) were merged (c). Under similar conditions, **2d** (2x) treated *E. faecalis* cells were photographed for DAPI (d) and PI (e), and the images were merged using Image (f).

To further explore the mechanism by which the compound targeted the bacterial cells, fluorescent microscopy was performed on the bacterial cells treated with the compound. Post **2d** treatment, *E. faecalis,* and *S. aureus* were treated with two fluorescent dyes 4′,6-diamidino-2-phenylindole (DAPI), and propidium iodide (PI). DAPI is a fluorescent dye that binds to DNA and emits blue fluorescence upon excitation by ultraviolet light and PI is also a fluorescent dye that binds to DNA, but it emits red fluorescence upon excitation by ultraviolet light. PI is a membrane-impermeable DNA binding fluorescent dye whereas DAPI is a membrane-permeable DNA binding dye. PI only enters cells with compromised membranes and stains the nucleic acid of dead cells. DAPI is permeable to the cell wall and fluoresces upon binding to chromosomal DNA, whether the cells are dead or alive. For control, the cells were treated with DAPI and PI without prior treatment with compound **2d**. The DAPI/PI staining results revealed the presence of red fluorescence in both *S. aureus* and *E. faecalis* bacterial strains. The red fluorescence observed in the bacterial cells indicates the uptake and binding of PI, suggesting compromised cell membrane integrity. This red fluorescence was visually distinguishable from the blue fluorescence emitted by DAPI, which stains the DNA of both live and dead bacteria. The identification of compromised membrane integrity is of significance as it can indicate the susceptibility of *S. aureus* and *E. faecalis* strains to antimicrobial treatments. The presence of red fluorescence provides evidence of cell damage and potential loss of viability, suggesting that these strains may be more susceptible to the effects of **2d** targeting the cell membrane. From Figures 5A,B, it is evident that the antibacterial activity of compound **2d** was due to damage to the cell wall of the bacterial cells. These findings suggest that compound **2d** targets the cell wall of bacterial cells, leading to compromised membrane integrity and ultimately cell death.

Conducting cell membrane-related studies on synthesized derivatives is a reasonable approach to understand their mechanisms of action. The cell membrane plays a critical role in regulating the passage of molecules and ions in and out of the cell, as well as in cell signalling and communication. Therefore, studying how the synthesized derivatives interact with the cell membrane can provide valuable insights into their potential mechanisms of action. However, we understand it is important to note that cell membrane-related studies alone may not provide a comprehensive understanding of the mechanisms of action of the synthesized derivatives. There could be additional mechanisms involved in their effects on cell viability that are independent of the cell membrane but DAPI and PI are commonly used fluorescent dyes that can be used to assess cell viability and membrane integrity. DAPI stains the nuclei of both live and dead cells, while PI is excluded from live cells but stains the DNA of dead or damaged cells with compromised membrane integrity. DAPI/PI staining allows for the differentiation between live cells (DAPI-positive, PI-negative). By using DAPI/PI staining, we wanted to assess the overall cell viability and determine whether the synthesized derivative have any detrimental effects on cell membrane integrity, which may lead to cell death.

#### 3.2.6 Biofilm inhibition assay

The compound **2d**, with the strong antibacterial activity, was tested against American Type Culture Collection (ATCC) strains of biofilm-forming Gram (+) bacteria, *S. aureus, E. faecalis, B. cereus* and Gram (−) bacteria, *E. coli, P. aeruginosa, K. pneumonia*. The Quantitative assessment of biofilm inhibition was done by calculating the percentage inhibition of the compound through spectrophotometric analysis for each bacterial strain by Microtiter Plate Biofilm Assay. The Qualitative assessment was done by CV staining and visualization under the microscope.

The percentage of biofilm inhibition was determined at various concentrations of compound **2d** (0.25–128 μg/mL), and the values reported are the means of three optical density (OD) measurements to ensure more reliable and accurate results. Compound **2d** exhibited significant biofilm inhibitory activity against all tested bacterial strains in a concentration-dependent manner. The percentage of biofilm inhibition varied across different concentrations and bacterial species. For *S. aureus*, compound **2d** displayed increasing biofilm inhibition percentages with higher concentrations. At the highest tested concentration of 128 μg/mL, the biofilm inhibition was 70.30%. Similarly, for *E. faecalis*, the biofilm inhibition reached 73.04% at the same concentration. *Bacillus cereus* showed a distinct trend, with biofilm inhibition percentages of 61.23% and 53.09% at the highest concentration (128 μg/mL) and half that concentration (64 μg/mL), respectively. However, the inhibitory effect decreased significantly at lower concentrations. Among the Gram (−) bacteria, *Pseudomonas aeruginosa* demonstrated substantial biofilm inhibition, with percentages ranging from 62.07% at 128 μg/mL to 35.47% at 0.25 μg/mL. *Escherichia coli* and *K. pneumonia* exhibited lower biofilm inhibition percentages, ranging from 19.73% to 49.41% and from 14.07% to 45.59%, respectively, across the tested concentrations. Overall, these results indicate that compound **2d** possesses significant biofilm inhibitory activity against a range of biofilm-forming bacterial strains with the most significant inhibitory potential against *S. aureus* and *E. faecalis.*

**FIGURE 6:**
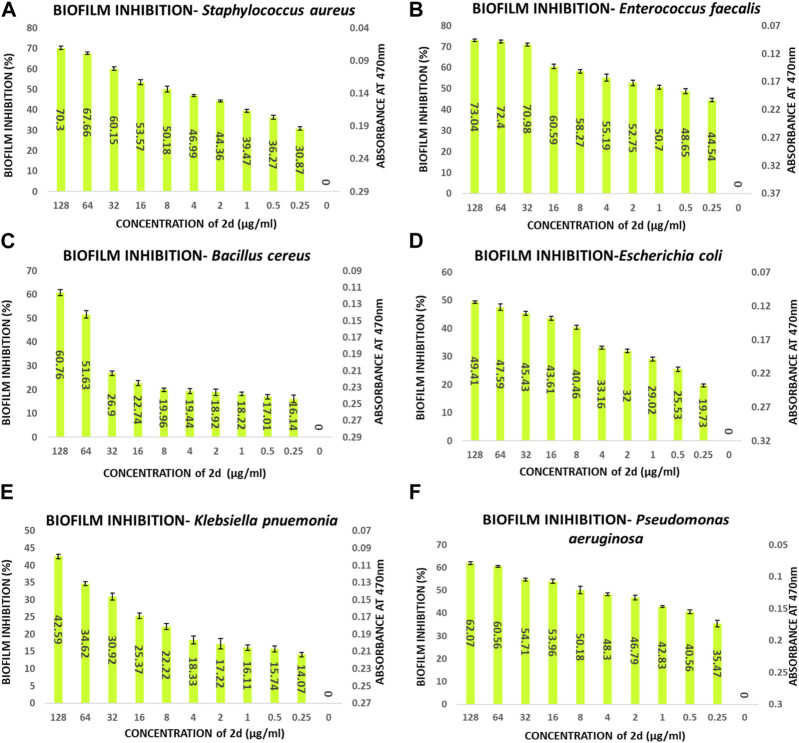
Quantitative analysis of biofilm percentage inhibition*-* **(A)** *Staphylococcus aureus;* **(B)** *E. faecalis;* **(C)** *Bacillus cereus;* **(D)** *E. coli;* **(E)** *Pseudomonas aeruginosa;* **(F)** *Klebsiella pneumonia.*

**FIGURE 7:**
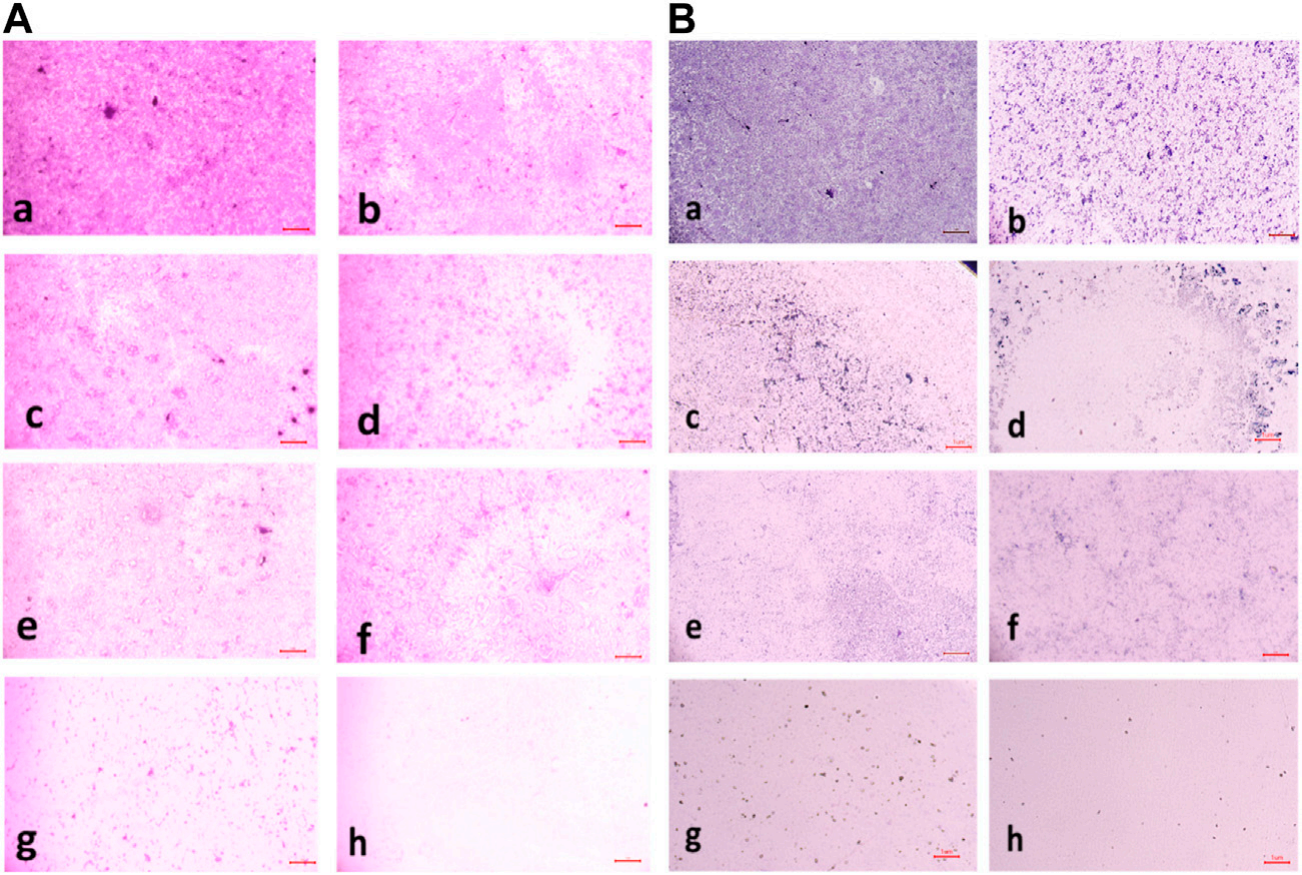
**(A)** Light microscopic images of biofilm (10x- Magnus) in *Staphylococcus aureus* at (a) Growth Control (b) 4 μg/mL (c) 8 μg/mL (d) 16 μg/mL (e) 32 μg/Ml (f) 64 μg/mL (g) 128 μg/mL treated wells (h) media control; **(B)** Light microscopic images of biofilm (10x- Magnus) in *E. faecalis* at (a) Growth Control (b) 4 μg/mL (c) 8 μg/mL (d) 16 μg/mL (e) 32 μg/mL (f) 64 μg/mL (g) 128 μg/mL treated wells (h) media control.

**FIGURE 8:**
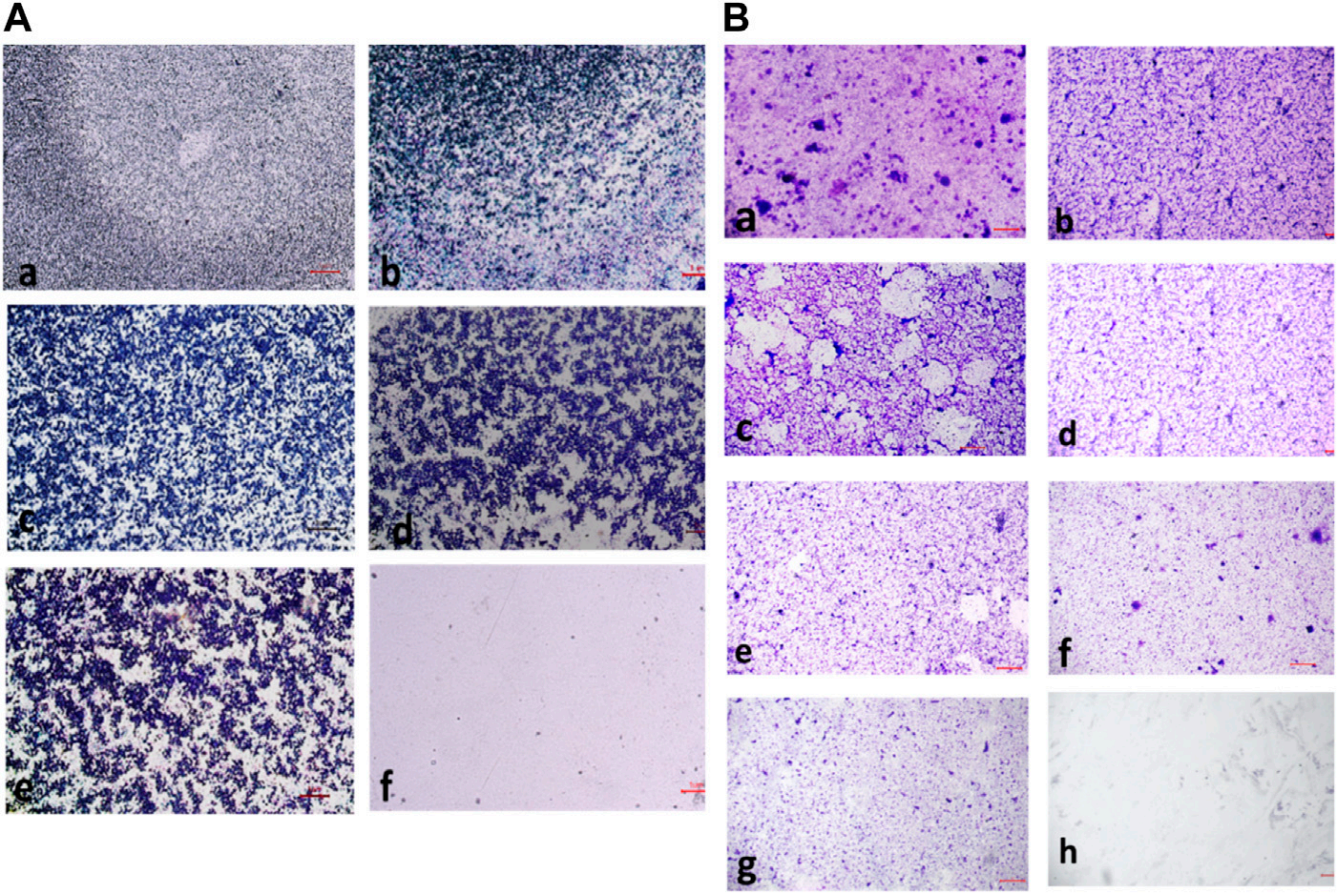
**(A)** Light microscopic images of biofilm (10x- Magnus) in *Bacillus cereus* at (a) Growth Control (b) 4 μg/mL (c) 16 μg/mL (d) 32 μg/mL (e) 128 μg/mL (f) media control; **(B)** Light Microscopic Images of Biofilm (10x- Magnus) in *E. coli* at (a) Growth Control (b) 4 μg/mL (c) 8 μg/mL (d) 16 μg/mL (e) 32 μg/mL (f) 64 μg/mL (g) 128 μg/mL treated wells (h) media control.

**FIGURE 9:**
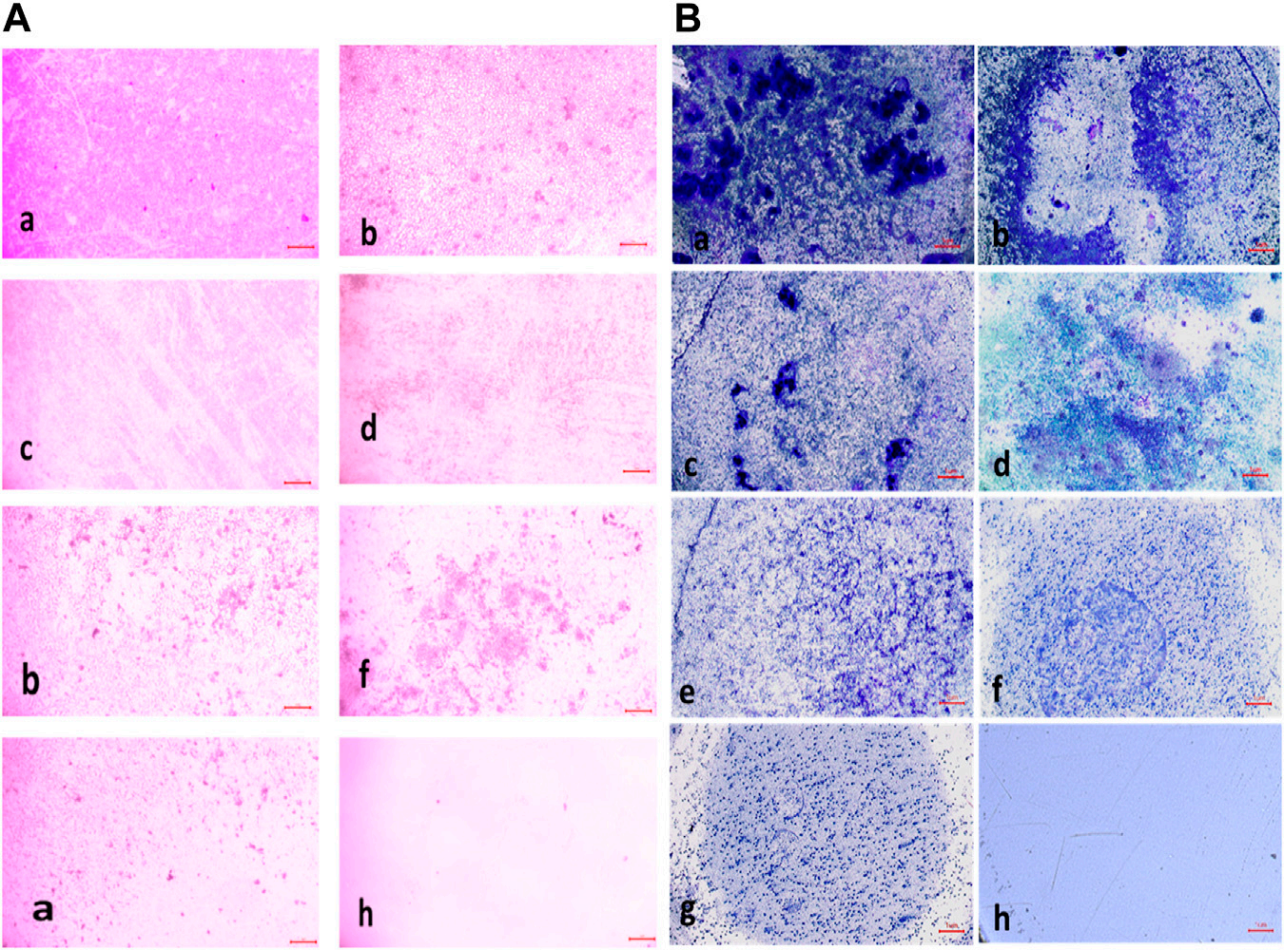
**(A)** Light Microscopic Images of Biofilm (10x- Magnus) in *Pseudomonas aeruginosa* at (a) Growth Control (b) 4 μg/mL(c) 8 μg/mL (d) 16 μg/mL (e) 32 μg/mL (f) 64 μg/mL (g) 128 μg/mL treated wells (h) media control; **(B)** Light Microscopic Images of Biofilm (10x- Magnus) in *Klebsiella pneumonia* at (a) Growth Control (b) 4 μg/mL(c) 8 μg/mL (d) 16 μg/mL (e) 32 μg/mL (f) 64 μg/mL (g) 128 μg/mL treated wells (h) media control.

The percentage of biofilm inhibition at different concentrations of compound **2d** was consistent with the qualitative analysis conducted using light microscopy. Microscopic examination revealed disrupted biofilm architecture and reduced biofilm biomass in the presence of compound **2d**, supporting the quantitative findings. The microscopic analysis provided visual confirmation of the biofilm inhibitory potential of compound **2d**. The growth control revealed a thick and compact biofilm structure, indicating the successful formation of mature biofilms by the tested bacterial strains. In contrast, the microscopic images of the biofilms treated with compound **2d** at different concentrations displayed notable differences compared to the growth control. The biofilms exposed to compound **2d** exhibited reduced biomass and disrupted biofilm architecture. The qualitative analysis corroborated the quantitative results, confirming that compound **2d** has a significant impact on biofilm formation across different bacterial strains. The visual evidence of reduced biomass and disrupted biofilm architecture shown in the figures at various concentrations supports the notion that compound **2d** interferes with the crucial steps of biofilm development (Figures 6–9).

#### 3.2.7 Biofilm eradication assay

The compound **2d** exhibited strong biofilm inhibition potential against both *E. faecalis* and *S. aureus* that prompted the evaluation of Biofilm Eradication potential against these two strains. The antibiofilm effect of the compound was evaluated both qualitatively and quantitatively by Light Microscopy and Crystal Violet method at concentrations 8, 16, 32, 64, and 128 μg/mL to evaluate the effect of the compound on various stages of biofilm formed at 24, 48 and 72 h. Light microscopic images revealed that the growth control wells exhibited significant biofilm formation at all three-time points. At 24 h, cell attachment was observed, followed by cell aggregation at 36 h, and extensive biofilm formation or mat formation at 48 h. However, in wells incubated with compound **2d** at different concentrations, non-aggregation of cells was observed during all stages of biofilm formation for both bacterial strains. This indicates the compound’s ability to inhibit cell aggregation, a critical step in biofilm development.

**FIGURE 10:**
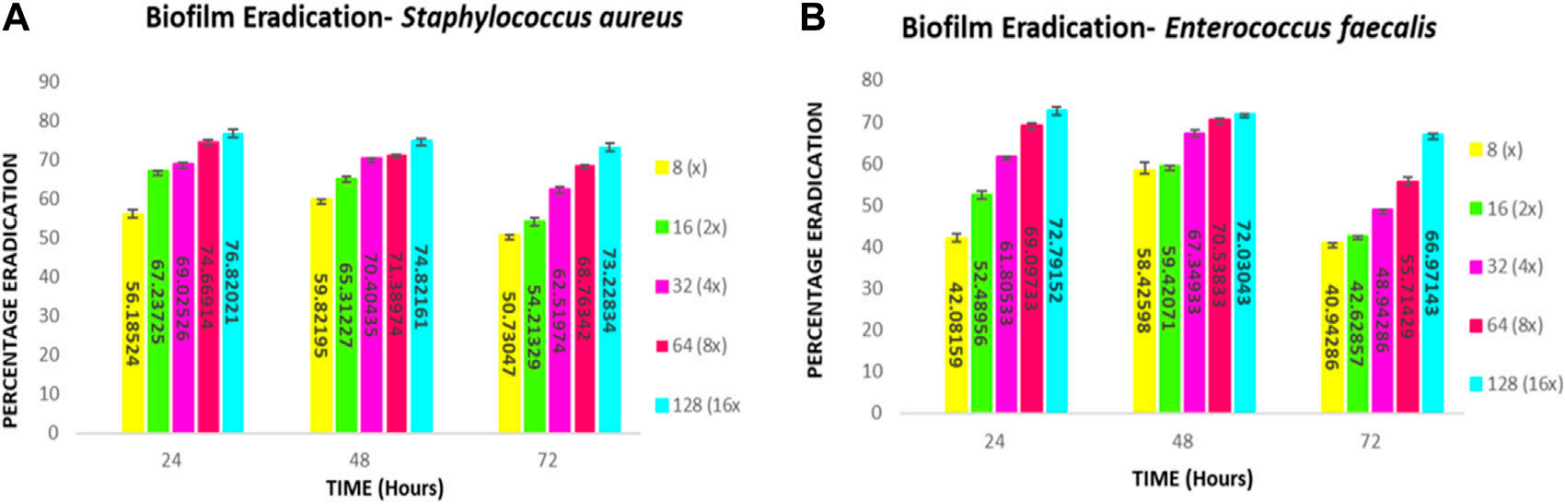
Quantitative Analysis: Biofilm eradication percentage **(A)** *Staphylococcus aureus* **(B)** *E. faecalis* at different concentrations of compound **2d** effecting the preformed 24-, 48- and 72-h biofilms.

This qualitative finding was further investigated with the crystal violet staining method by comparing the absorbance between the growth control wells and the wells incubated with the compound. The percentage of biofilm Eradication at various concentrations is shown (Figures 10A, B) which indicates a strong antibiofilm potential of the compound eradicating up to more than 40% of the biofilm at all concentrations. The crystal violet staining method confirmed the qualitative findings, demonstrating the compound’s antibiofilm potential. The absorbance measurements showed that compound **2d** eradicated over 40% of the biofilm at all tested concentrations. Notably, at higher concentrations, the compound exhibited substantial eradication potential against both *E. faecalis* and *S. aureus*, eliminating 70%–77% of the preformed biofilm at all three-time points. These results suggest that compound **2d** has a strong biofilm eradication capability. The qualitative and quantitative evaluation of biofilm eradication correlated with each other. The eradication potential of the compound on the densely pre-biofilm at 72 h against both bacterial strains indicates that the compound can be a promising agent for biofilm eradication. The quantitative evaluation of the percentage of biofilm eradication was done as per the formula mentioned in the methodology and the results represented are the mean values of the three OD readings taken for each concentration in different wells. The compound effectively inhibited cell aggregation and eradicated preformed biofilms in a dose-dependent manner.

The biofilm eradication percentages of compound **2d** against 24-h formed *S. aureus* biofilms were found to be 59% at 8 μg/mL, 67% at 16 μg/mL, 69% at 32 μg/mL, 74% at 64 μg/mL, and 76% at 128 μg/mL. For 48-h biofilms, the eradication percentages were 59%, 65%, 70%, 71%, and 74% at the corresponding concentrations. In the case of mature biofilms formed for 72 h, compound **2d** exhibited eradication percentages of 50%, 54%, 62%, 68%, and 73% at the respective concentrations.

The biofilm eradication percentages of compound **2d** against 24-h formed *E. faecalis* biofilms were found to be 42% at 8 μg/mL, 52% at 16 μg/mL, 61% at 32 μg/mL, 69% at 64 μg/mL, and 72% at 128 μg/mL. For 48-h biofilms, the eradication percentages were 58%, 59%, 67%, 70%, and 72% at the corresponding concentrations. In the case of mature biofilms formed for 72 h, compound **2d** exhibited eradication percentages of 40%, 42%, 48%, 55%, and 66% at the respective concentrations.

**FIGURE 11:**
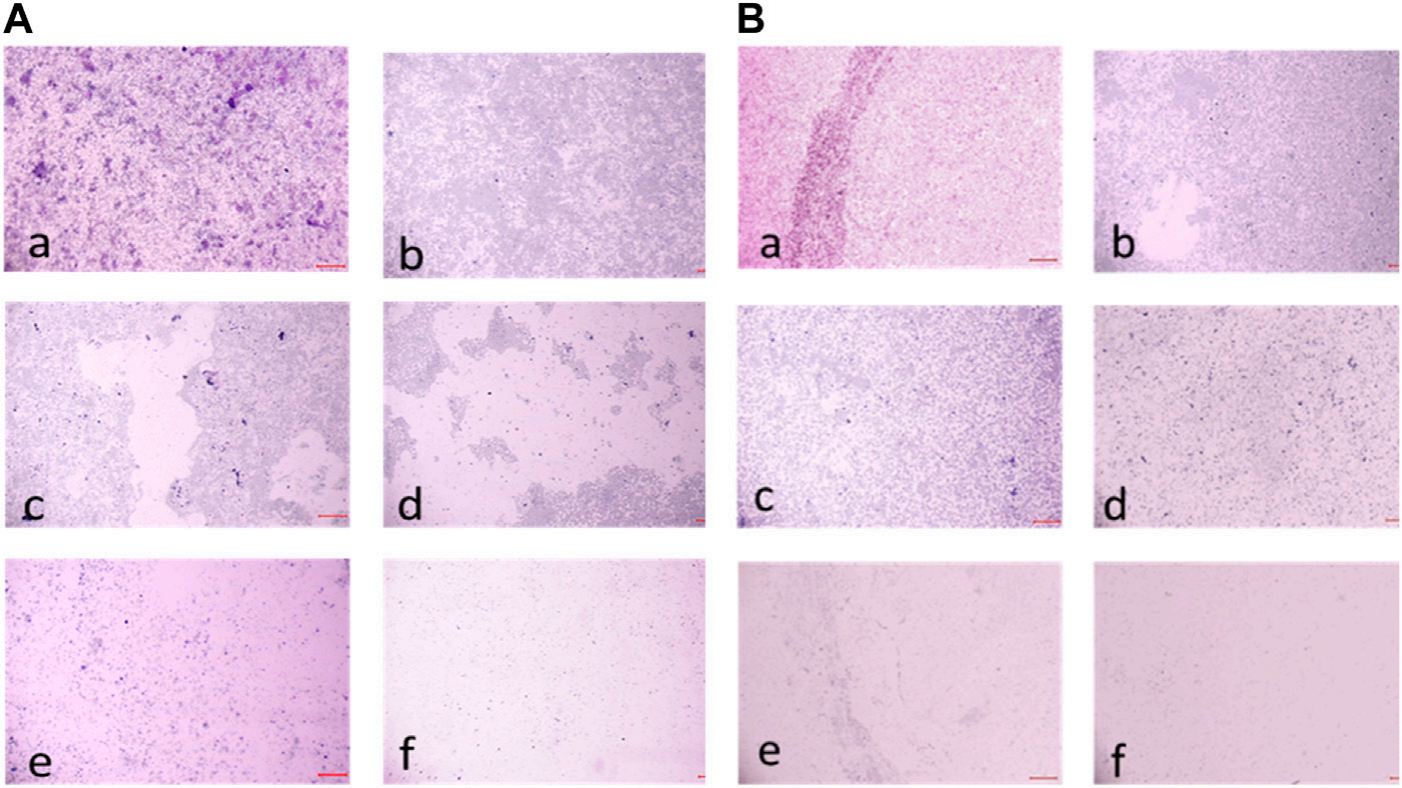
**(A)** Light microscopic images (20X) of biofilm eradication in 24 hour-preformed biofilm of *Staphylococcus aureus* by compound **2d**, (a) Growth control-showing initial attachment to surface (devoid of compound exhibiting biofilm formation after 24 h) (b) 8 μg/mL (c) 16 μg/mL (d) 32 μg/mL (e) 64 μg/mL (f) 128 μg/mL; **(B)** Biofilm of *Staphylococcus aureus* by compound 2d, (a) Growth control-showing aggregation of cells (devoid of compound exhibiting biofilm formation after 48 h) (b) 8 μg/mL (c) 16 μg/mL (d) 32 μg/mL (e) 64 μg/mL (f) 128 μg/mL.

**FIGURE 12:**
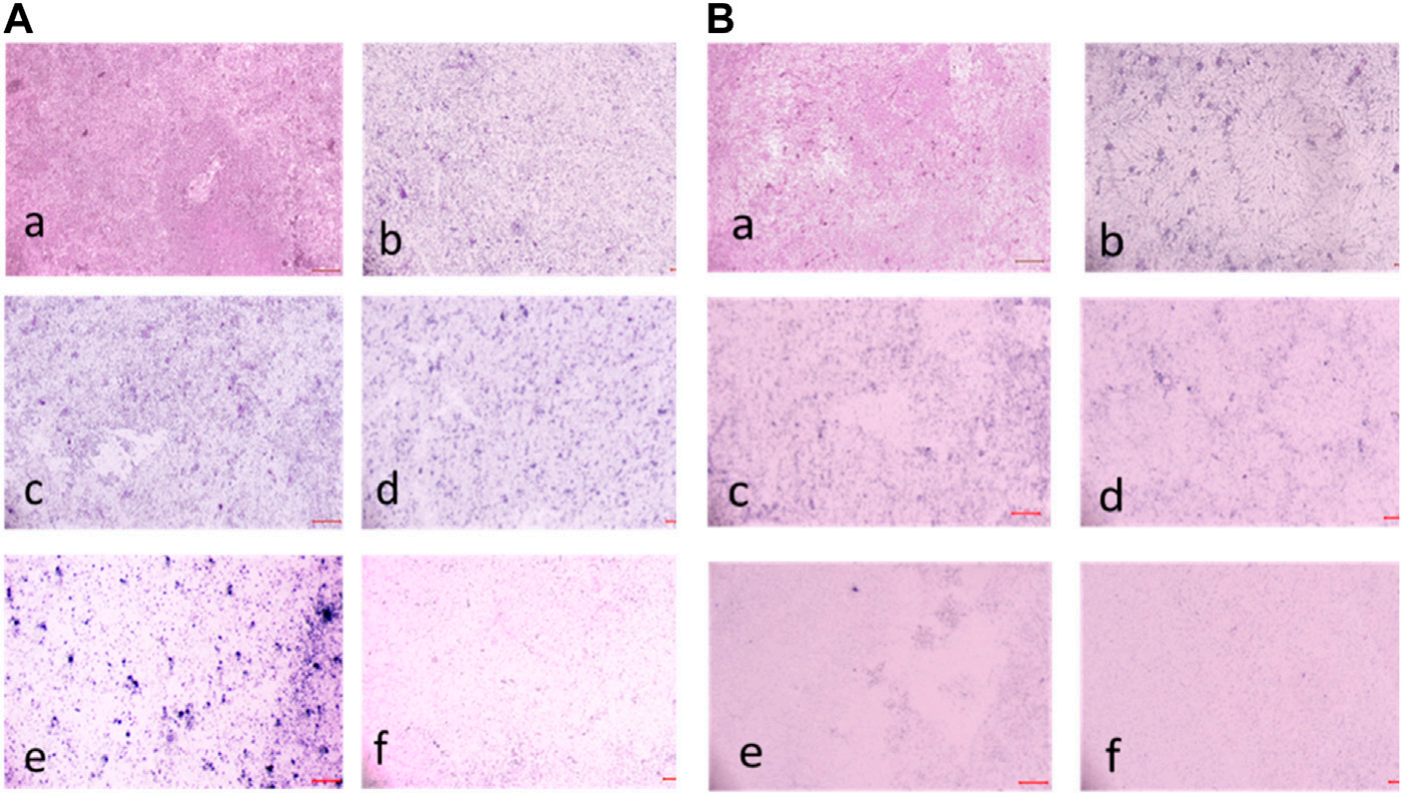
**(A)** Light Microscopic images (20X) of biofilm eradication in 72 hour-preformed biofilm of *S. aureus* by compound **2d**, (a) Growth control-showing mat formation (devoid of compound exhibiting biofilm formation after 72 h) (b) 8 μg/mL (c) 16 μg/mL (d) 32 μg/mL (e) 64 μg/mL (f) 128 μg/mL. **(B)** Light Microscopic images (20X) of biofilm eradication in 24 hour-preformed biofilm of *E. faecalis* by compound **2d**, (a) Growth control-showing initial attachment to surface (devoid of compound exhibiting Biofilm Formation after 24 h) **(**b) 8 μg/mL (c) 16 μg/mL (d) 32 μg/mL (e) 64 μg/mL (f) 128 μg/mL.

**FIGURE 13:**
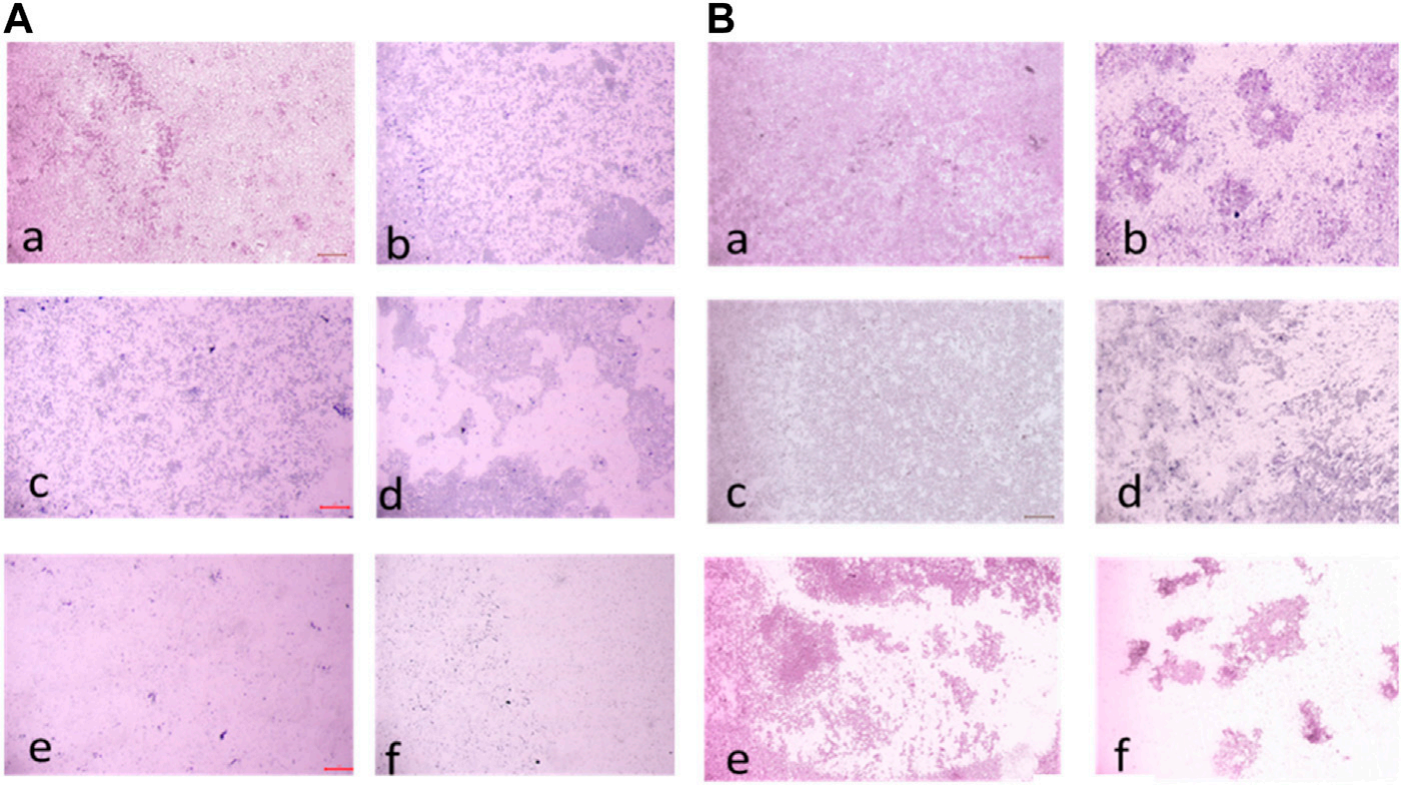
**(A)** Light microscopic images (20X) of biofilm eradication in 48 hour-preformed biofilm of *E. faecalis* by compound **2d**, (a) Growth control-showing aggregation of cells (devoid of compound exhibiting biofilm formation after 48 h) 8 μg/mL (c) 16 μg/mL (d) 32 μg/mL (e) 64 μg/mL (f) 128 μg/mL. **(B)** Light microscopic images (20X) of biofilm eradication in 72 hour-preformed biofilm of *E. faecalis* by compound **2d**, (a) Growth control-showing mat formation (devoid of compound exhibiting biofilm formation after 72 h) (b) 8 μg/mL (c) 16 μg/mL (d) 32 μg/mL (e) 64 μg/mL (f) 128 μg/mL.

These values represent the average mean of three independent experiments. The results of this study demonstrate the potent antibiofilm activity of compound **2d** against *S. aureus* and *E. faecalis* biofilms. The compound exhibited remarkable efficacy in eradicating biofilms at various time points and concentrations. Compound **2d** effectively eradicated a significant proportion of the 24-h formed biofilms. Even at the lowest concentration tested (8 μg/mL), the compound achieved a substantial eradication percentage. As the concentration increased the eradication percentages also increased, reaching 72%–76% at the highest concentration (128 μg/mL). These findings highlight the concentration-dependent efficacy of compound **2d** in disrupting and eliminating early-stage *S. aureus* and *E. faecalis* biofilms. Furthermore, compound **2d** demonstrated consistent efficacy in eradicating biofilms formed over 48 h. The eradication percentages indicated its ability to penetrate and disrupt biofilm structures at various concentrations. Notably, even at lower concentrations, the compound achieved eradication percentages above 50%–65%, demonstrating its potency against established biofilms in both bacteria. Compound **2d** also exhibited substantial efficacy in eradicating mature biofilms formed over 72 h. The eradication percentages ranged from 50% to 73% for *S. aureus* and 40%–66% for *E. faecalis*, indicating its ability to target and disrupt the mature biofilm matrix. These results suggest that compound **2d** possesses strong biofilm-eradicating properties, even in the presence of a mature biofilm structure. The findings from this study highlight the potential of compound **2d** as an effective antibiofilm agent against *S. aureus* and *E. faecalis*. The ability of the compound to significantly reduce biofilm density and disrupt biofilm architecture is consistent with the qualitative observations made during the study. The strong correlation between the qualitative and quantitative evaluations supports the robustness of the antibiofilm activity of compound **2d**. The compound effectively inhibited cell adhesion, auto-aggregation, and mat formation, as observed under light microscopy. These findings suggest that compound **2d** may target key stages of biofilm development and inhibit biofilm formation (Figures 11–13).

#### 3.2.8 Cytotoxicity assay

**FIGURE 14:**
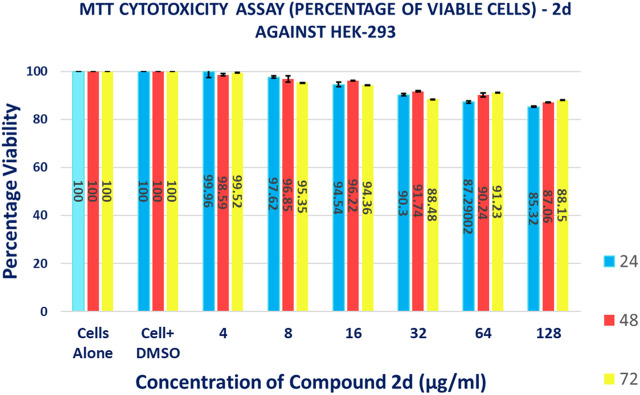
Graphical representation of the results of MTT cytotoxicity assay.

The MTT-based cell viability assay performed on HEK-293 verified that cells exposed to **2d** compound at varying concentrations for different time durations have a non-toxic effect on the human embryonic kidney cell lines. The results of the MTT-based cell viability assay were conducted on HEK-293 cells. Surprisingly, the findings indicate that the cells exposed to **2d** exhibited a non-toxic response, demonstrating higher percentages of viable cells even at higher concentrations and longer exposure durations (Figure 14). This suggests that the **2d** compound is cytocompatible and does not have a concentration- or time-dependent effect on cell viability. The MTT assay revealed that cell viability was significantly higher even at higher concentration ranges of 16–128 μg/mL. The percentage viability was calculated using formula (5).

The data presented in Table 6 demonstrate the favorable impact of the **2d** compound on HEK-293 cell viability. At the lowest concentration tested (4 μg/mL), the average viability percentages remained consistently high across all concentration and time point combinations. At the highest concentration tested (128 μg/mL), the average viability percentage after 72 h of exposure was 88.15% ± 0.005774%, indicating that even at this relatively elevated concentration, the **2d** compound did not significantly compromise cell viability. This trend of high viability percentages persisted throughout the experiment, irrespective of the exposure duration. It is important to note that these results are specific to HEK-293 cells and should not be generalized to other cell types without further experimentation.

**TABLE 6:** Percentage of HEK-293 cell viability after treatment with different concentrations 2d for different time points (percentages expressed are the average values of three experiments ±SD).

Average percentage of viable cells after exposure with 2d for different time durations			
Conc. (µg/mL)	24 h	48 h	72 h
**4**	99.96 ± 2.532	98.59 ± 0.501	99.52 ± 0.001
**8**	97.62 ± 0.475	96.85 ± 1.269	95.35 ± 0.010017
**16**	94.54 ± 0.989	96.22 ± 0.173	94.36 ± 0.006429
**32**	90.30 ± 0.557	91.74 ± 0.289	88.48 ± 0.002309
**64**	87.29 ± 0.428	90.24 ± 0.765	91.23 ± 0.024664
**128**	85.32 ± 0.164	87.06 ± 0.153	88.15 ± 0.005774

## 4 Conclusion, scope and limitations

In summary, we successfully synthesized 2-azidobenzothiazoles by employing sodium nitrite and sodium azide in a brief reaction time and established a straightforward, effective, and high-yielding method. The promising antibacterial activity of benzothiazole derivatives prompted us to perform *in vitro* antibacterial activity of the synthesized compounds. The experimental results revealed that compound **2d** exhibited potential antibacterial activity against various bacterial strains. As a result, compound **2d** could be exploited as a potential antimicrobial target for many drug-resistant microorganisms.

Our comprehensive *in vitro* study holds significant implications and contributions to the scientific community. It addresses the issue of antibiotic resistance which is fuelled by the decline in the development of new antibiotics that necessitates the exploration of novel therapeutic agents. Our study provides valuable insights into the antibacterial activity of 2-azidobenzothiazoles, demonstrating their potency against both Gram (+) and Gram (−) bacteria, including drug-resistant strains. Moreover, biofilm-associated infections pose a significant challenge in clinical settings due to their inherent resistance to conventional antibiotics. Our study reveals the remarkable ability of the lead compound **2d**, to inhibit and eradicate biofilms formed by various bacterial strains. This finding has important implications for developing effective strategies to manage chronic and persistent infections associated with biofilms. The identification of a potent lead compound **2d**, with selective cytotoxicity towards bacterial cells while sparing human cells, highlights its potential for therapeutic applications. This finding offers promising prospects for the development of safe and effective antimicrobial treatments. Further optimization and exploration of the lead compound, along with other compounds in the series, may lead to the development of novel antibiotics or antimicrobial agents. Our study further exemplifies the importance of drug discovery efforts in combating antibiotic resistance and biofilm-associated infections.

Although our study provides valuable insights into the antibacterial and antibiofilm potential, it is essential to acknowledge some limitations. Firstly, our experiments were conducted *in vitro,* and further investigations should include *in vivo* models to assess the compound’s efficacy and safety in more complex biological systems. Our experiments were conducted solely *in vitro*, using laboratory-based assays. While these provide a controlled environment for evaluating the compound’s efficacy, they may not fully represent the complexity of bacterial infections *in vivo*. Further investigations using animal models or clinical studies are necessary to validate the potential therapeutic applications of these compounds. Although we performed a comprehensive analysis, focusing on multiple aspects such as MIC, MBC, kill kinetics, post-antibiotic effect, cytotoxicity, biofilm inhibition, and biofilm eradication, numerous other factors could influence the efficacy and safety of these compounds. Factors such as pharmacokinetics, tissue penetration, drug metabolism, and potential drug-drug interactions were not specifically addressed in our study. While our study identified one potent lead compound **2d** with significant antibacterial and antibiofilm activity, it is crucial to explore a broader range of compounds and structural modifications. The development of multiple lead compounds with diverse chemical scaffolds can increase the chances of finding more effective and selective antimicrobial agents. Our study focused primarily on *in vitro* assessments, and clinical data regarding the safety and efficacy of these compounds in human subjects are lacking. Clinical trials are needed to evaluate the compounds’ performance in real-world scenarios and assess factors such as dosage, treatment duration, and potential side effects. Addressing these limitations and conducting further research will enhance our understanding of the potential of 2-azidobenzothiazoles as antimicrobial agents and pave the way for their future development and clinical application.

## Data Availability

The original contributions presented in the study are included in the article/Supplementary Material, further inquiries can be directed to the corresponding authors.
