# Supplementary material for: Design, synthesis, and unraveling the antibacterial and antibiofilm potential of 2-azidobenzothiazoles: insights from a comprehensive in vitro study
Source: Front Chem. 2023 Sep 7;11:1264747. doi: 10.3389/fchem.2023.1264747 (PMC10513370; doi:10.3389/fchem.2023.1264747)
Supplement: Supplementary file 1 [file DataSheet1.pdf]

Electronic Supporting Information

Design, synthesis, and unraveling the antibacterial and antibiofilm potential of 2-azidobenzothiazoles: Insights from a comprehensive *in vitro* study

Tanzeela Qadir[1], Sadaat A. Kanth[2], Mohammad Aasif[3], Abdalla N. Fadul[4] , Gulam N. Yatoo[3], Kailash Jangid[5], Mushtaq A. Mir[4] , Wajahat A. Shah[6]*, and Praveen K. Sharma[1]*

[1]Department of Chemistry, School of Chemical Engineering and Physical Sciences, Lovely Professional University, Phagwara Punjab, India
[2]Centre of Research for Development & P.G Programme in Microbiology, School of Biological Sciences, University of Kashmir, Srinagar, J&K, India
[3]Department of Chemistry, National Institute of Technology, Hazratbal, Srinagar, J&K India
[4]Department of Clinical Laboratory Sciences, College of Applied Medical Science, King Khalid University, Saudi Arabia
[5]Department of Chemistry, Central University of Punjab, Bathinda, Punjab, India
[6]Laboratory of Natural product and Designing organic synthesis, Department of Chemistry, University of Kashmir, Srinagar, J&K, India

* **CORRESPONDENCE**: Praveen K. Sharma, pk_pandit1982@yahoo.com; praveen.14155@lpu.co.in; Wajahat A, Shah, doctorwajaht@gmail.com

Table of Contents

1. ^{1}H NMR spectra of the synthesized compounds..2
2. IR spectra of the synthesized compounds……………………………………………………10
3. MASS spectra of the compounds subjected..11
4. Antibacterial Activity………………………………………………………………………..18
4.1. Minimum Inhibitory Concentration……………………………………………………….18
4.2. Minimum Bactericidal Concentration………………………………………………………22
4.3. MTT based Time Kill Kinetics…………………………………………………………….25
4.4. Biofilm Inhibition Assay…………………………………………………………………....26
4.5 Cytotoxicity Assay…………………………………………………………………………..26

1. ^{1}H NMR spectra of the synthesized compounds

1.1. ^{1}H NMR of 2-Azido-4,6-difluorobenzothiazole (*2a*):

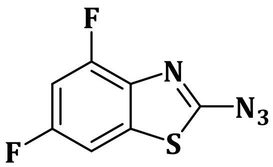

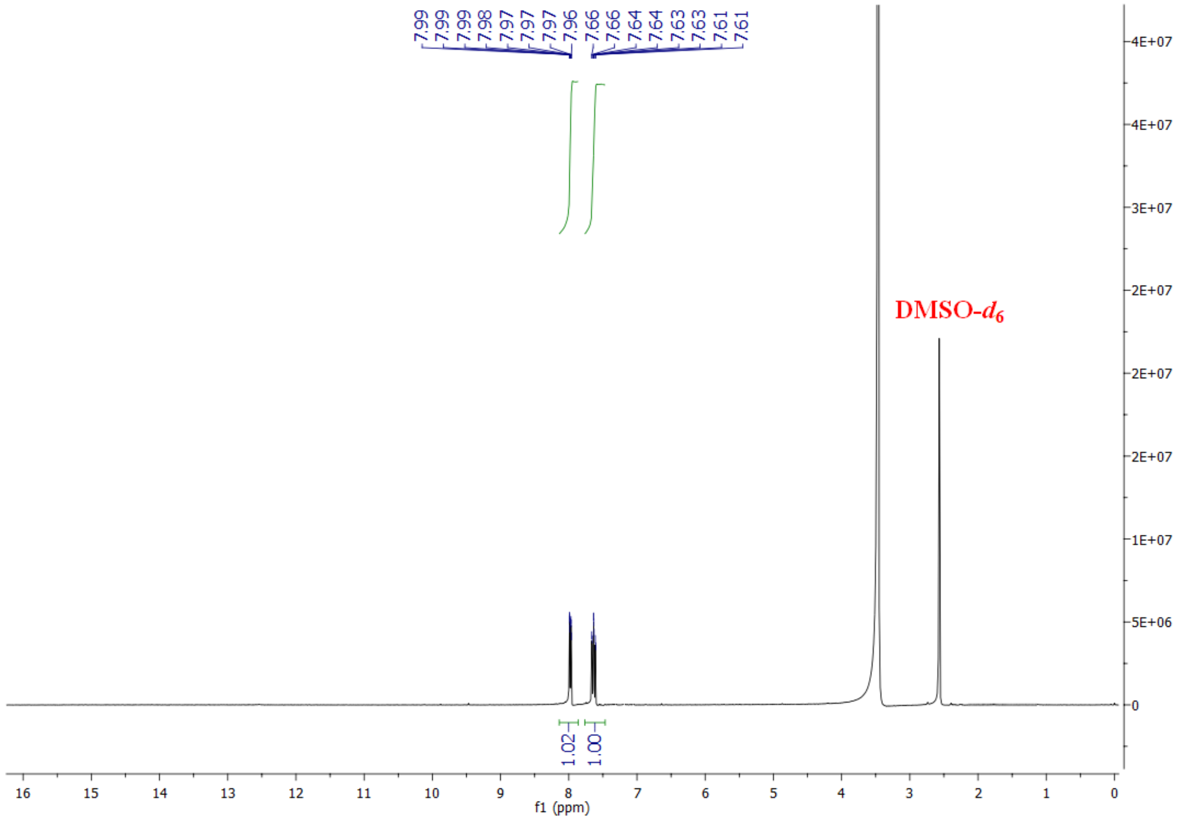

1.2. ^{1}H NMR of 2-Azido-6-methoxybenzothiazole (*2b*):

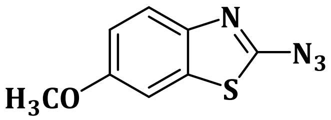

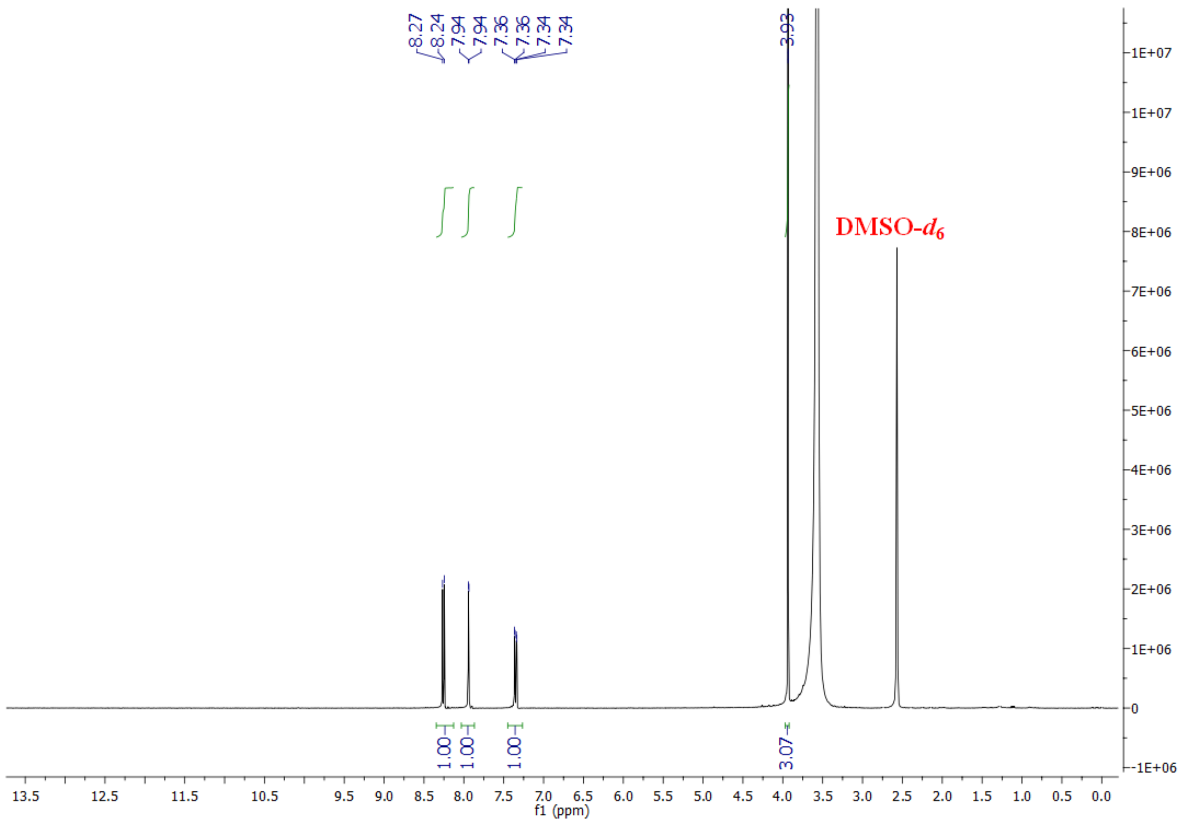

1.3. ^{1}H NMR of Ethyl 2-azidobenzothiazole-6-carboxylate (*2c*):

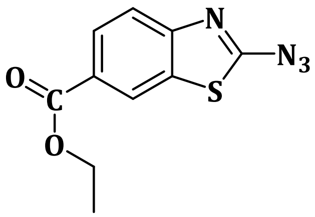

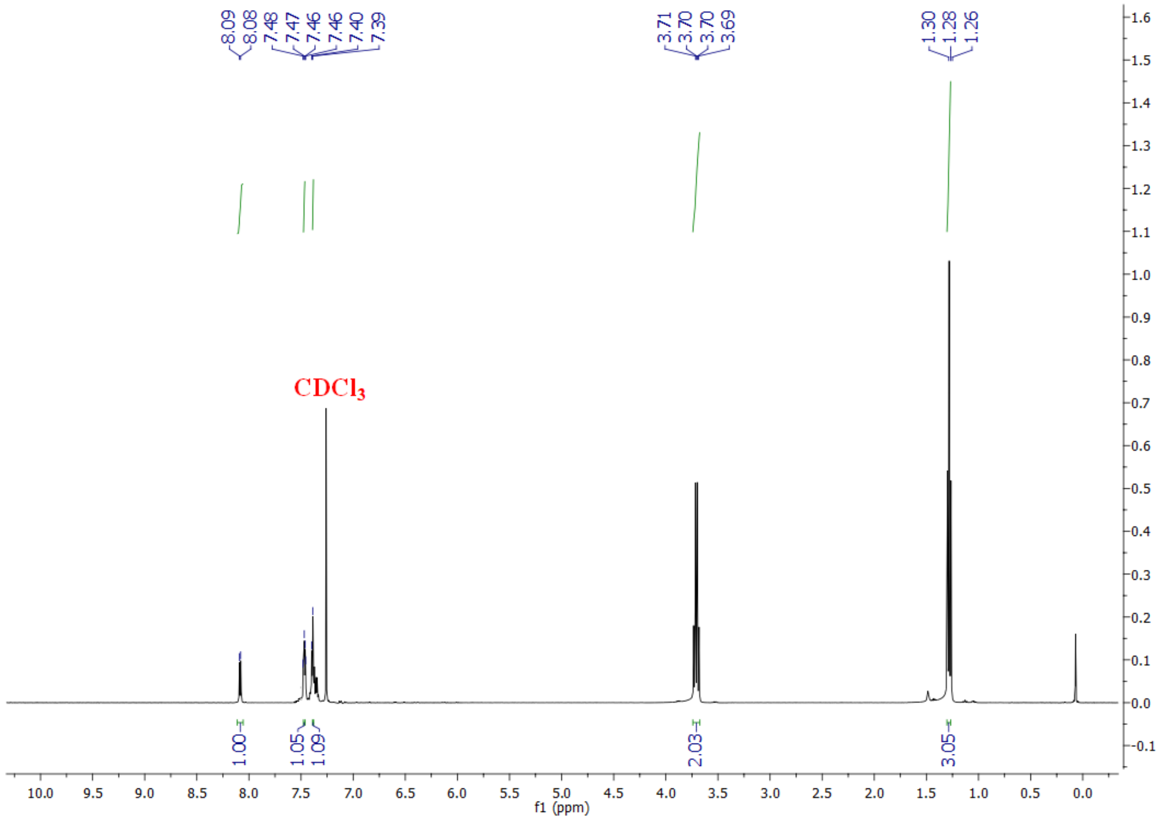

1.4. ^{1}H NMR of 2-Azido-6-nitro-benzothiaole (*2d*):

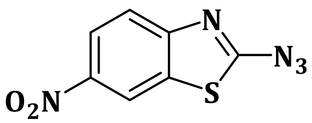

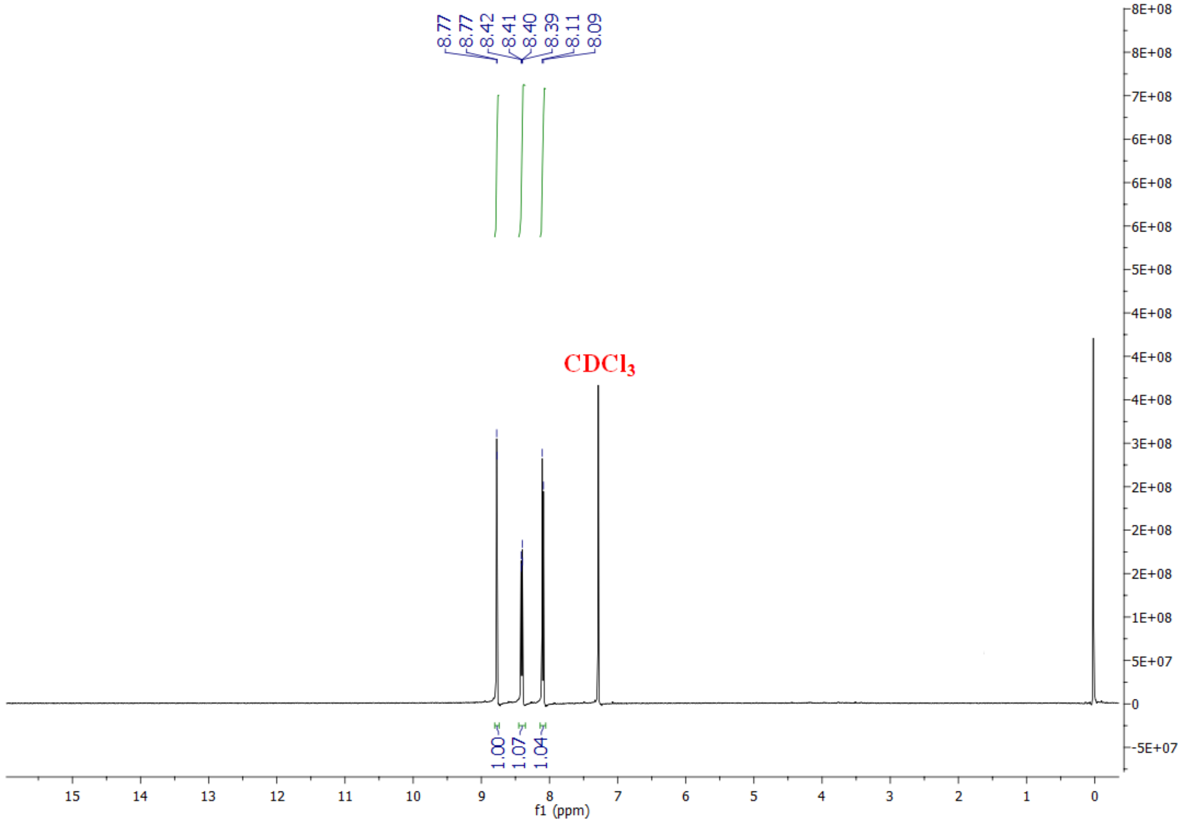

1.5. ^{1}H NMR of 2-Azidobenzothiazole (*2e*):

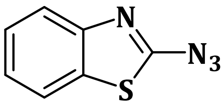

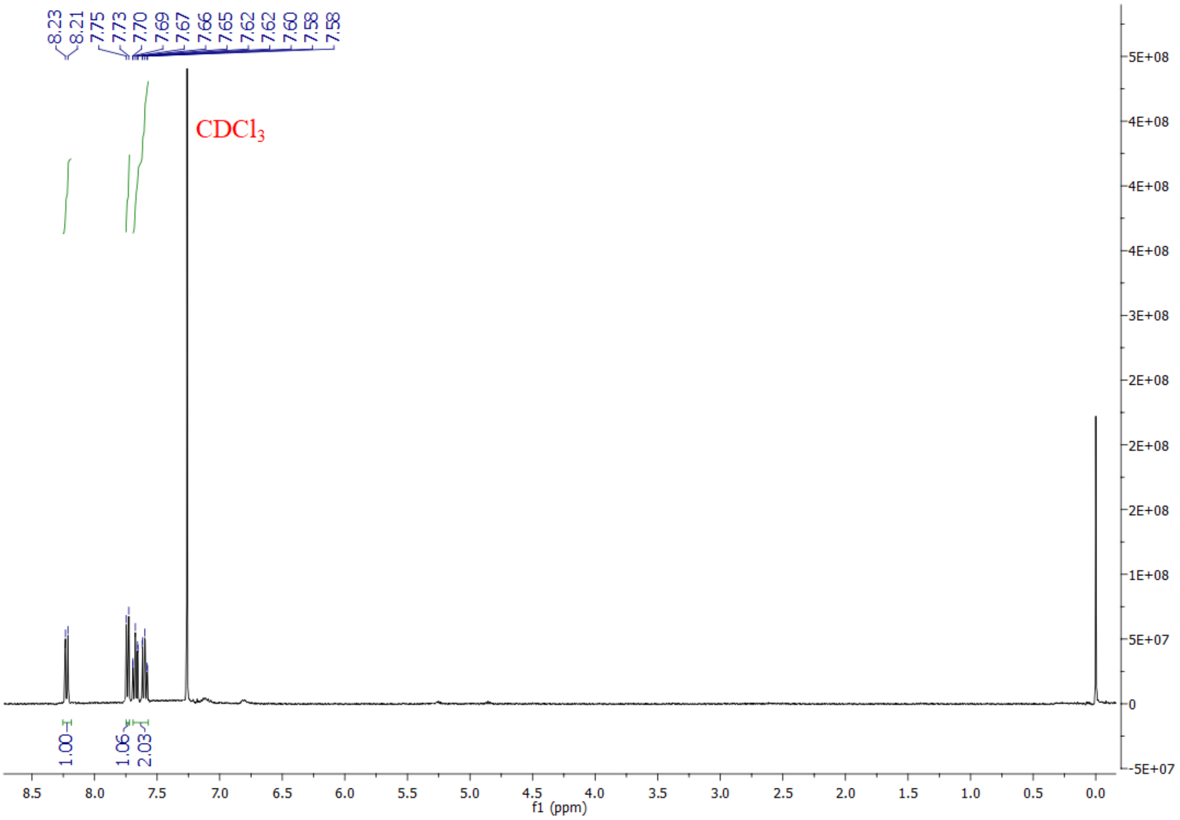

1.6. ^{1}H NMR of 2-Azido-4-methylbenzothiazole (*2f*):

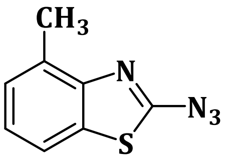

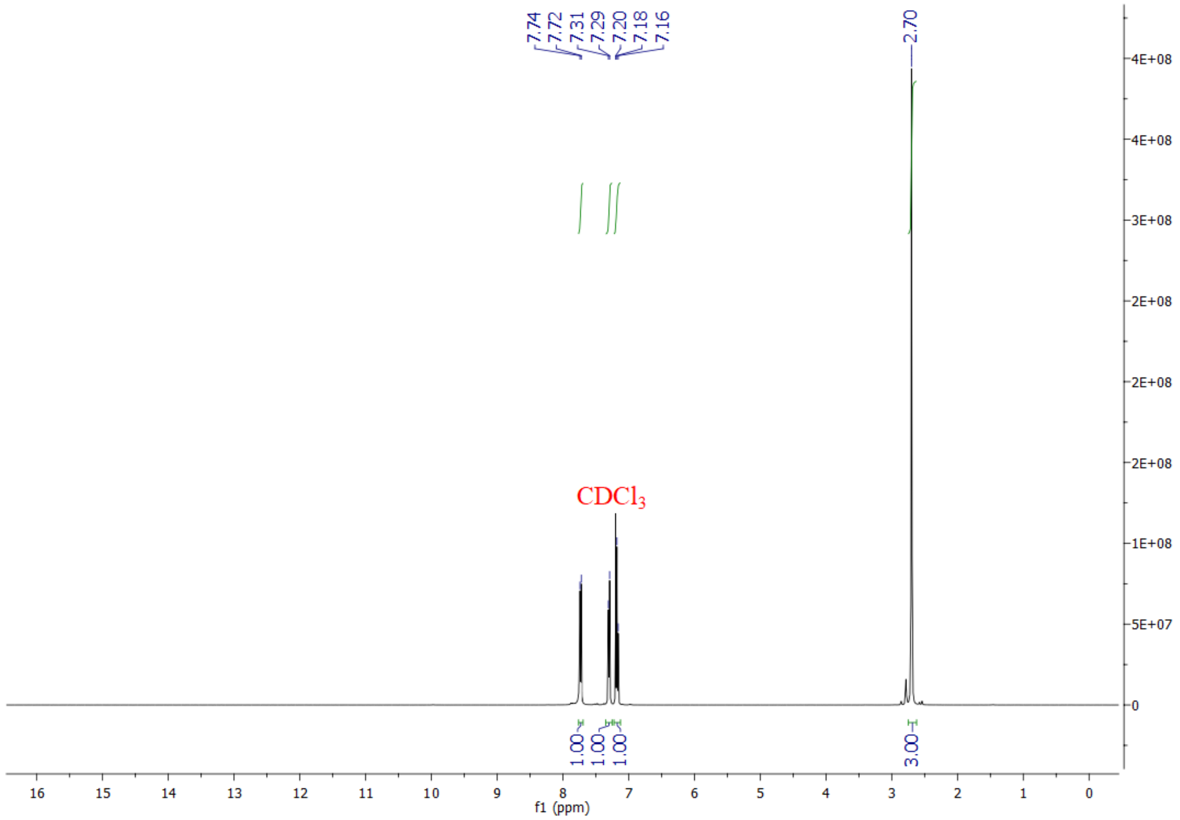

1.7. ^{1}H NMR of 2-Azido-6-ethoxybenzothiazole (*2g*):

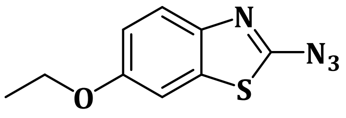

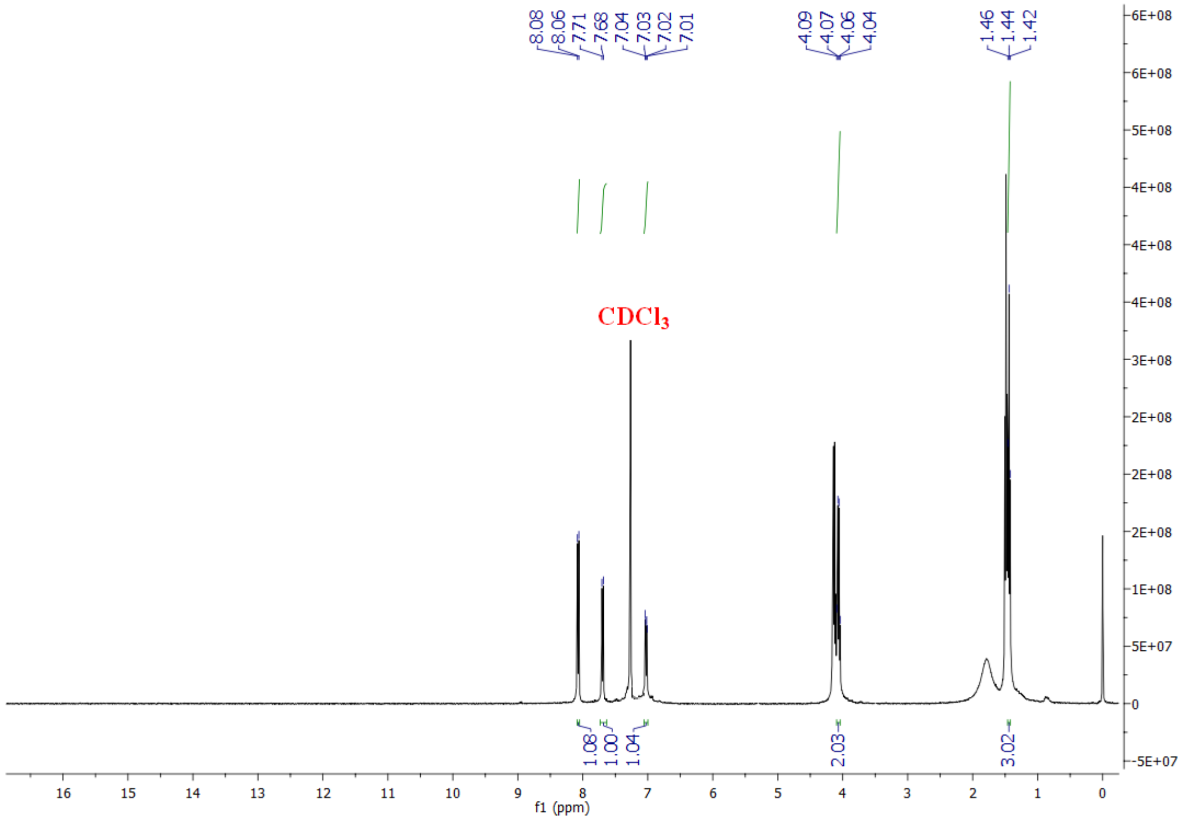

1.8. ^{1}H NMR of 2-Azido-5-bromobenzothiazole (*2h*):

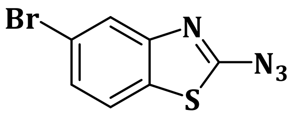

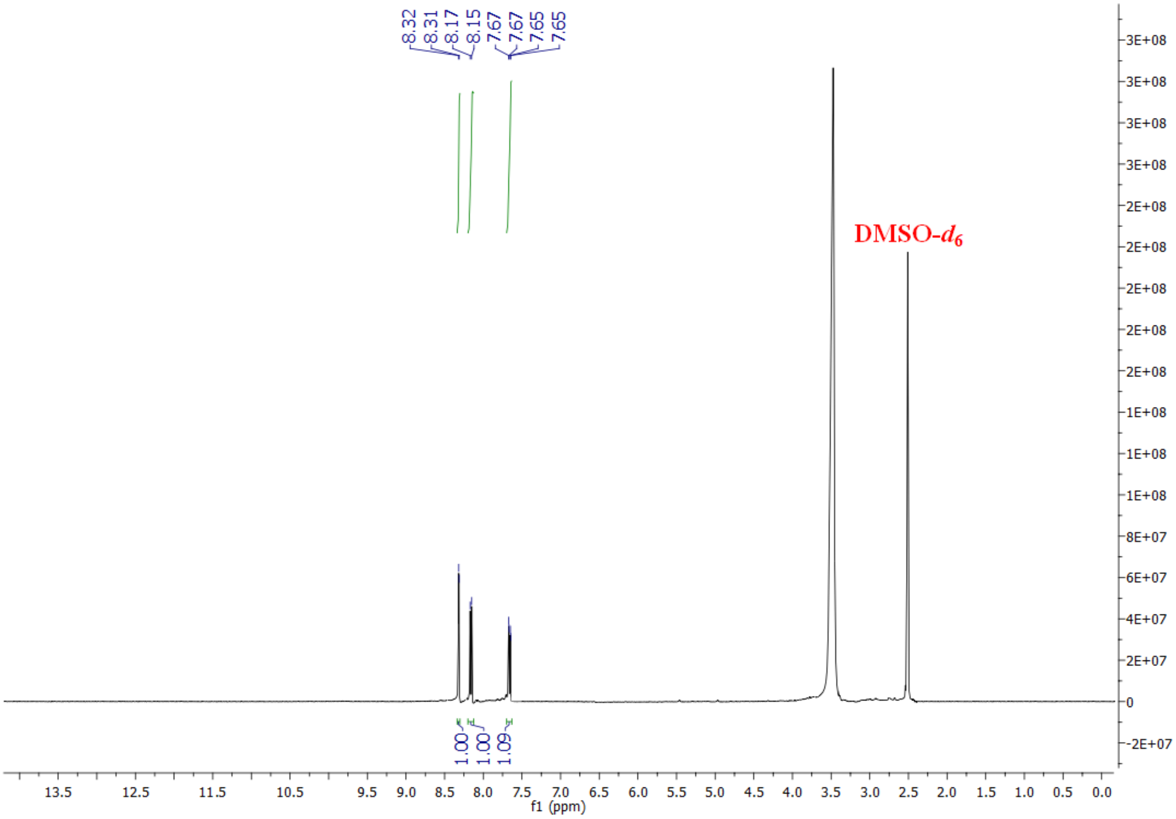
8.32
8.31
8.17
8.15
7.67
7.67
7.65
7.65
DMSO-d_6
1.00
1.00
1.09
f1 (ppm)

2. IR spectra of the synthesized compounds

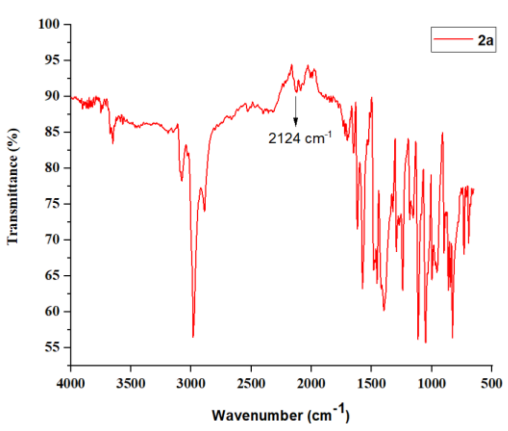

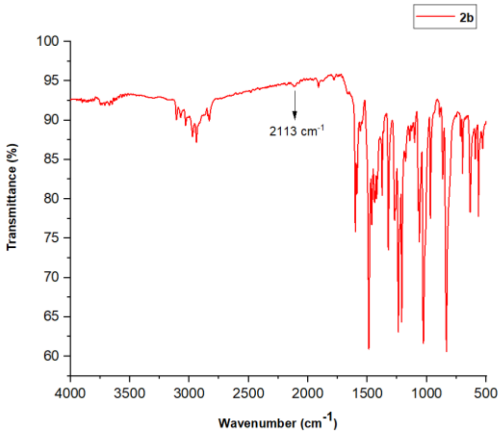

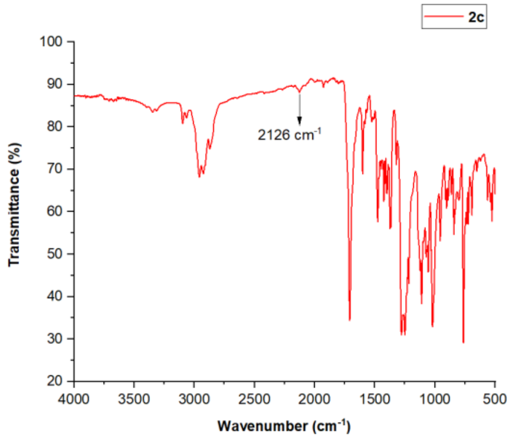

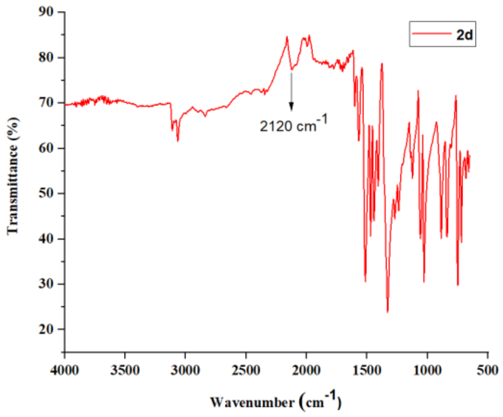

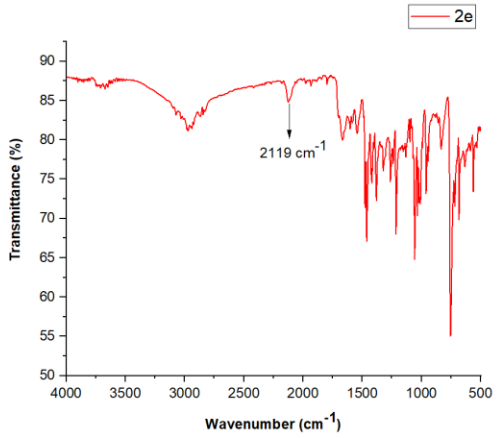

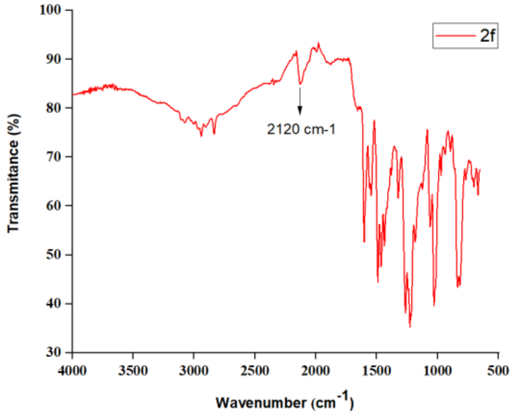

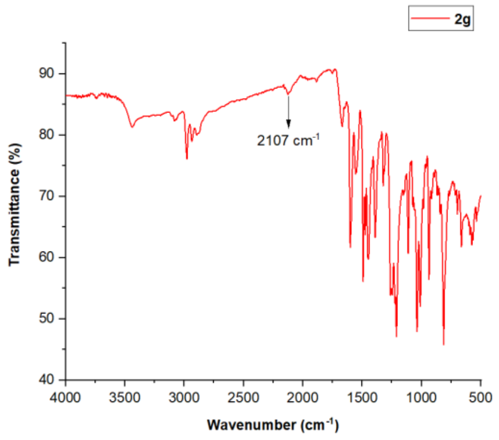

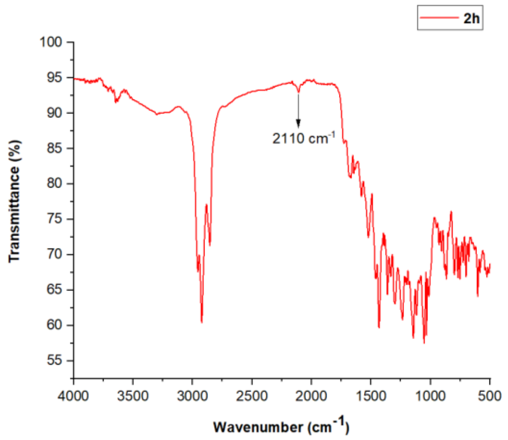

3. Mass spectra of the compounds subjected

3.1. 2-Azido-4,6-difluorobenzothiazole (*2a*):

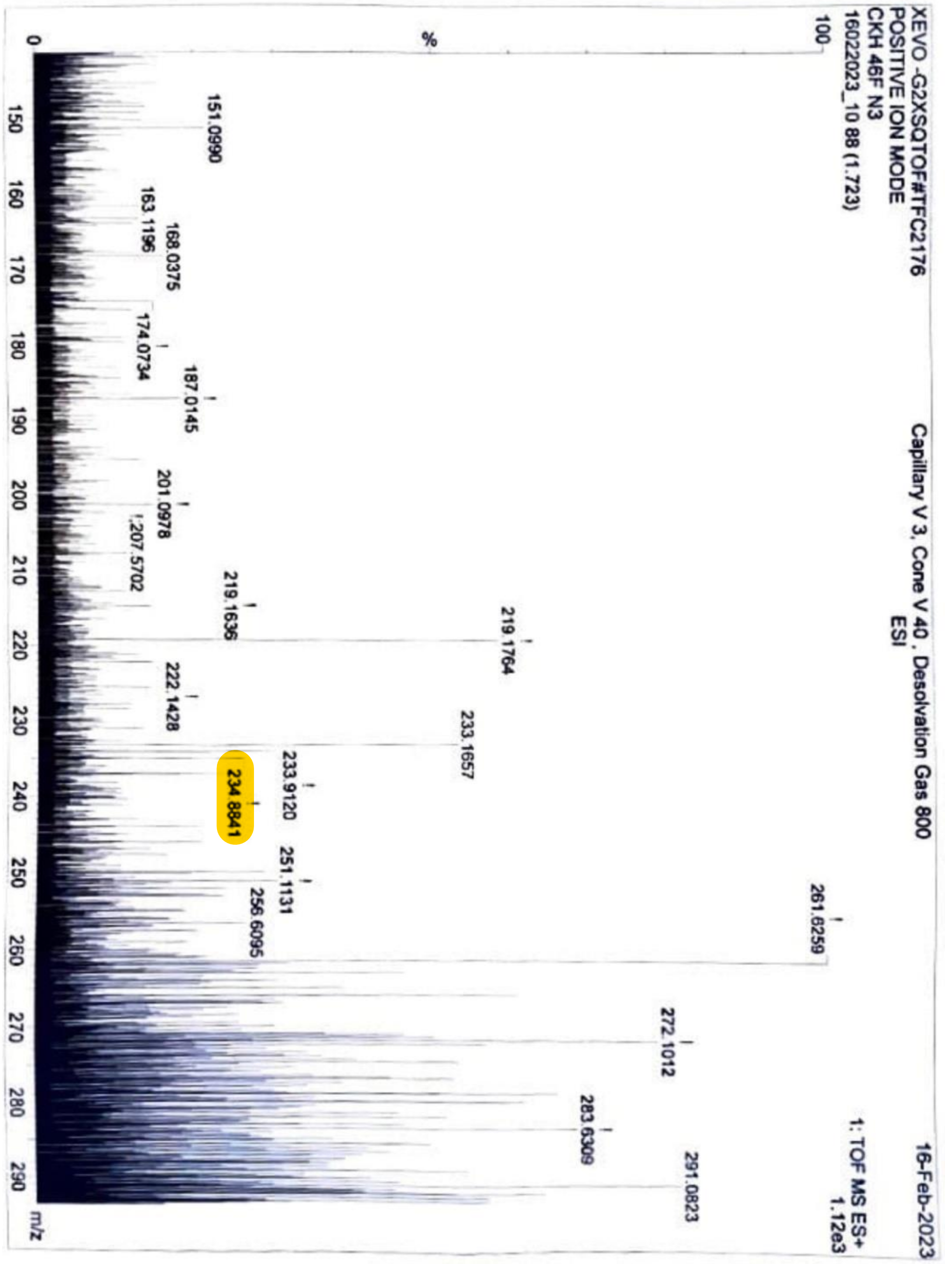

3.2. 2-Azido-6-methoxybenzothiazole (*2b*):

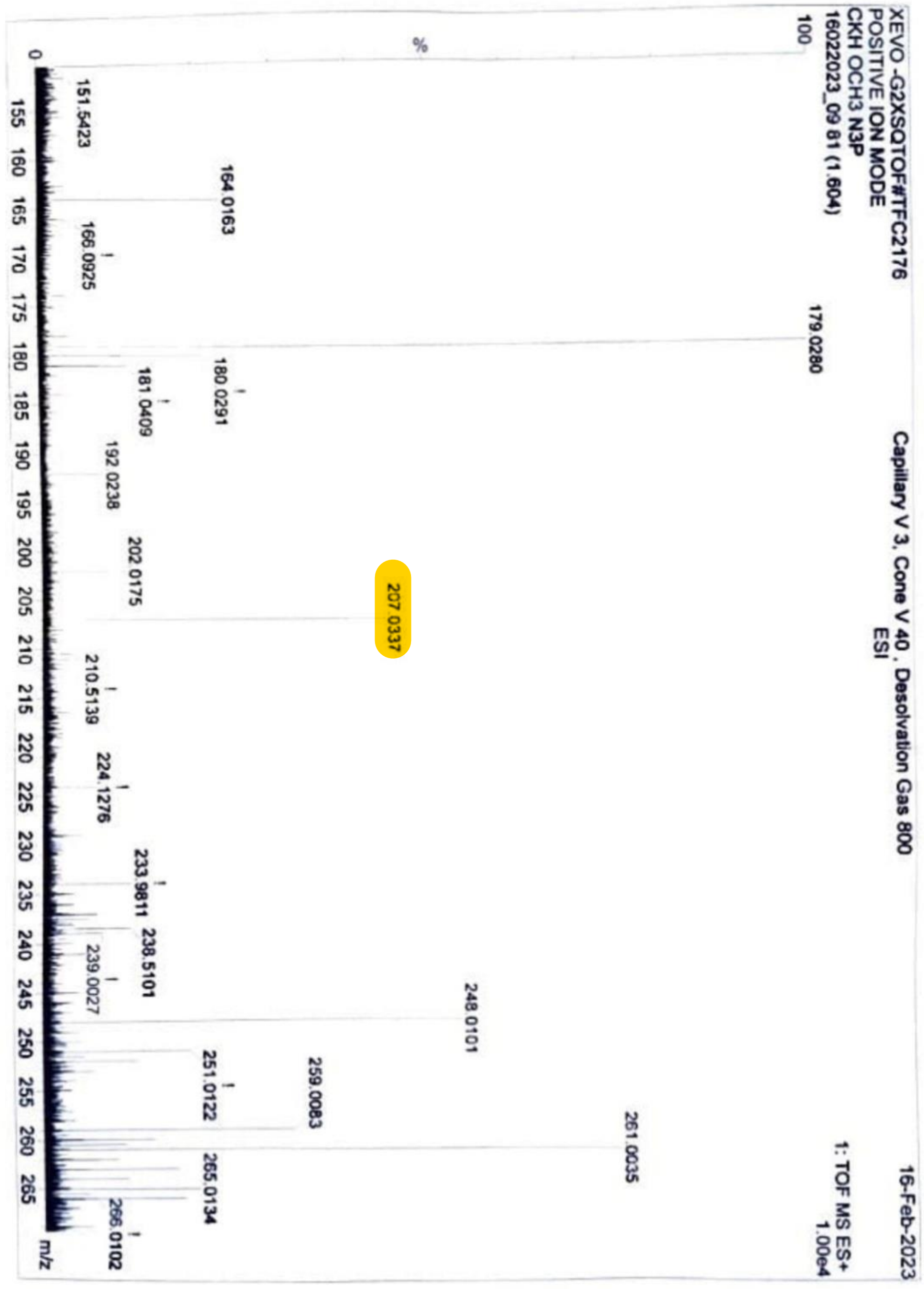
XEVO -G2XSQTOF#TFC2176
POSITIVE ION MODE
CKH OCH3 N3P
16022023_09 81 (1.604)
Capillary V 3, Cone V 40, Desolvation Gas 800
ESI
16-Feb-2023
1: TOF MS ES+
1.00e4
100
%
0
151.5423
164.0163
166.0925
179.0280
180.0291
181.0409
192.0238
202.0175
207.0337
210.5139
224.1276
233.9811
238.5101
239.0027
248.0101
251.0122
259.0083
261.0035
265.0134
266.0102
155 160 165 170 175 180 185 190 195 200 205 210 215 220 225 230 235 240 245 250 255 260 265
m/z

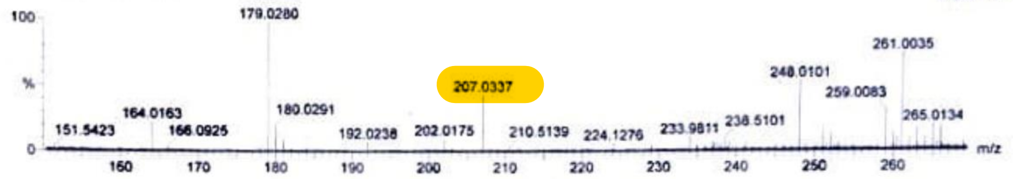

Single Mass Analysis
Tolerance = 5.0 PPM / DBE: min = -1.5, max = 50.0
Element prediction: Off
Number of isotope peaks used for i-FIT = 3

Monoisotopic Mass, Even Electron Ions
116 formula(e) evaluated with 1 results within limits (up to 10 best isotopic matches for each mass)
Elements Used:
C: 0-20 H: 0-15 N: 0-4 O: 0-3 Na: 0-1 S: 0-1
XEVO -G2XSQTOF#TFC2176
POSITIVE ION MODE
CKH OCH3 N3P
16022023_09 81 (1.604)

Capillary V 3, Cone V 40, Desolvation Gas 800
ESI

16-Feb-2023

1: TOF MS ES+
1.00e+004

Minimum:				-1.5				
Maximum:		5.0	5.0	50.0				
Mass	Calc. Mass	mDa	PPM	DBE	i-FIT	Norm	Conf(%)	Formula
207.0337	207.0341	-0.4	-1.9	7.5	1192.3	n/a	n/a	C8 H7 N4 O S

3.3. 2-Azido-6-nitrobenzothiazole (*2d*):

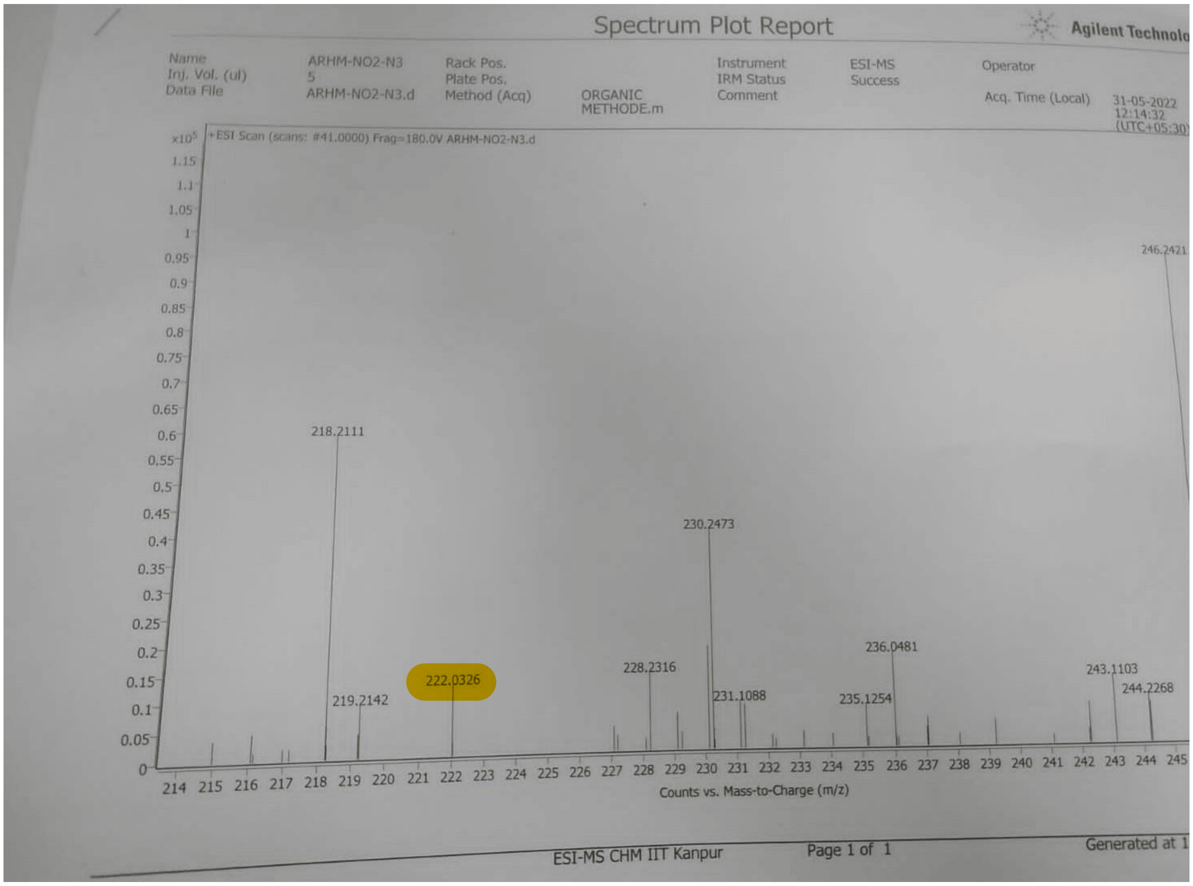

3.4. 2-Azidobenzothiazole (*2e*):

Sample Information

Analyzed by	: Admin
Analyzed	: 2/2/2023 11:30:21 AM
Sample Type	: Unknown
Level #	: 1
Sample Name	: KIJ-68
Sample ID	: KIJ-68
IS Amount	: [1]=1
Sample Amount	: 1
Dilution Factor	: 1
Vial #	: 1
Injection Volume	: 1.00
Data File	: E:\Mass Analysis\02-02-2023\KIJ-68.qgd
Org Data File	: E:\Mass Analysis\02-02-2023\KIJ-68.qgd
Method File	: D:\gc-ms\MEthod\DI_CUP.qgm
Org Method File	: D:\gc-ms\MEthod\DI_CUP.qgm
Report File	:
Tuning File	: C:\GCMSsolution\System\Tune1\17012023_N_CI-F1_Blind.qgt
Modified by	: Admin
Modified	: 2/2/2023 4:23:59 PM

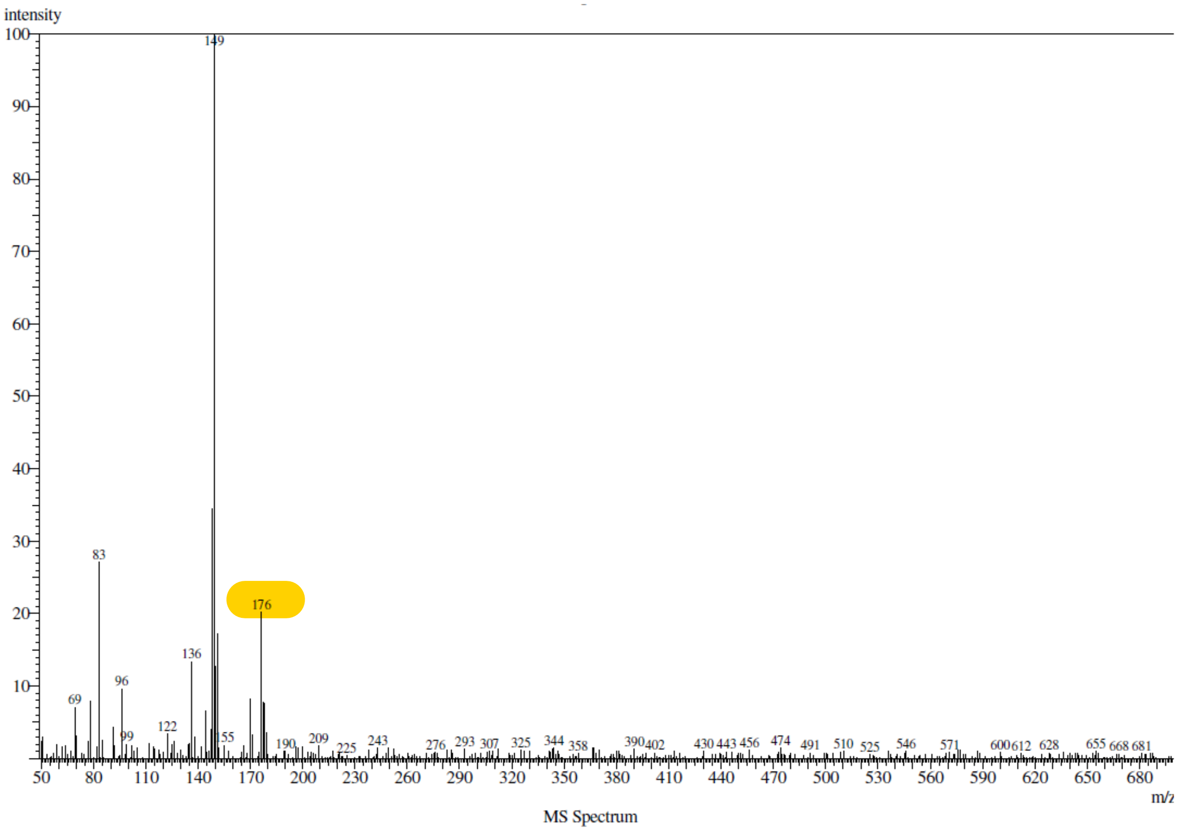

3.5. 2-Azido-4-methylbenzothiazole (*2f*):

Sample Information

Analyzed by	: Admin
Analyzed	: 2/2/2023 12:17:45 PM
Sample Type	: Unknown
Level #	: 1
Sample Name	: KIJ-67
Sample ID	: KIJ-67
IS Amount	: [1]=1
Sample Amount	: 1
Dilution Factor	: 1
Vial #	: 1
Injection Volume	: 1.00
Data File	: E:\Mass Analysis\02-02-2023\KIJ-67.qgd
Org Data File	: E:\Mass Analysis\02-02-2023\KIJ-67.qgd
Method File	: D:\gc-ms\MEthod\DI_CUP.qgm
Org Method File	: D:\gc-ms\MEthod\DI_CUP.qgm
Report File	:
Tuning File	: C:\GCMSsolution\System\Tune1\17012023_N_CI-F1_Blind.qgt
Modified by	: Admin
Modified	: 2/2/2023 4:24:23 PM

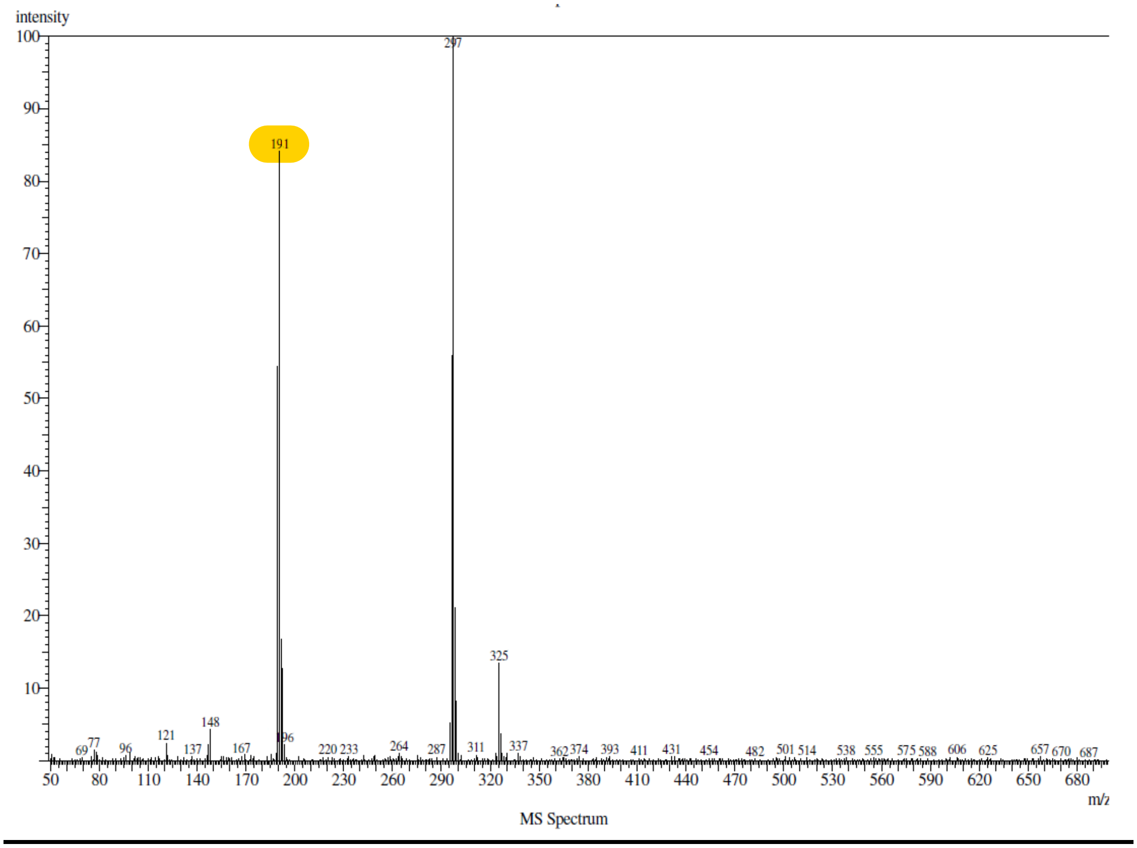

3.6. 2-Azido-6-ethoxybenzothiazole (*2g*):

Sample Information

Analyzed by	: Admin
Analyzed	: 2/2/2023 2:56:30 PM
Sample Type	: Unknown
Level #	: 1
Sample Name	: KIJ-66-1
Sample ID	: KIJ-66-1
IS Amount	: [1]=1
Sample Amount	: 1
Dilution Factor	: 1
Vial #	: 1
Injection Volume	: 1.00
Data File	: E:\Mass Analysis\02-02-2023\KIJ-66-1.qgd
Org Data File	: E:\Mass Analysis\02-02-2023\KIJ-66-1.qgd
Method File	: D:\gc-ms\MEthod\DI_CUP.qgm
Org Method File	: D:\gc-ms\MEthod\DI_CUP.qgm
Report File	:
Tuning File	: C:\GCMSsolution\System\Tune1\17012023_N_CI-F1_Blind.qgt
Modified by	: Admin
Modified	: 2/2/2023 4:24:48 PM

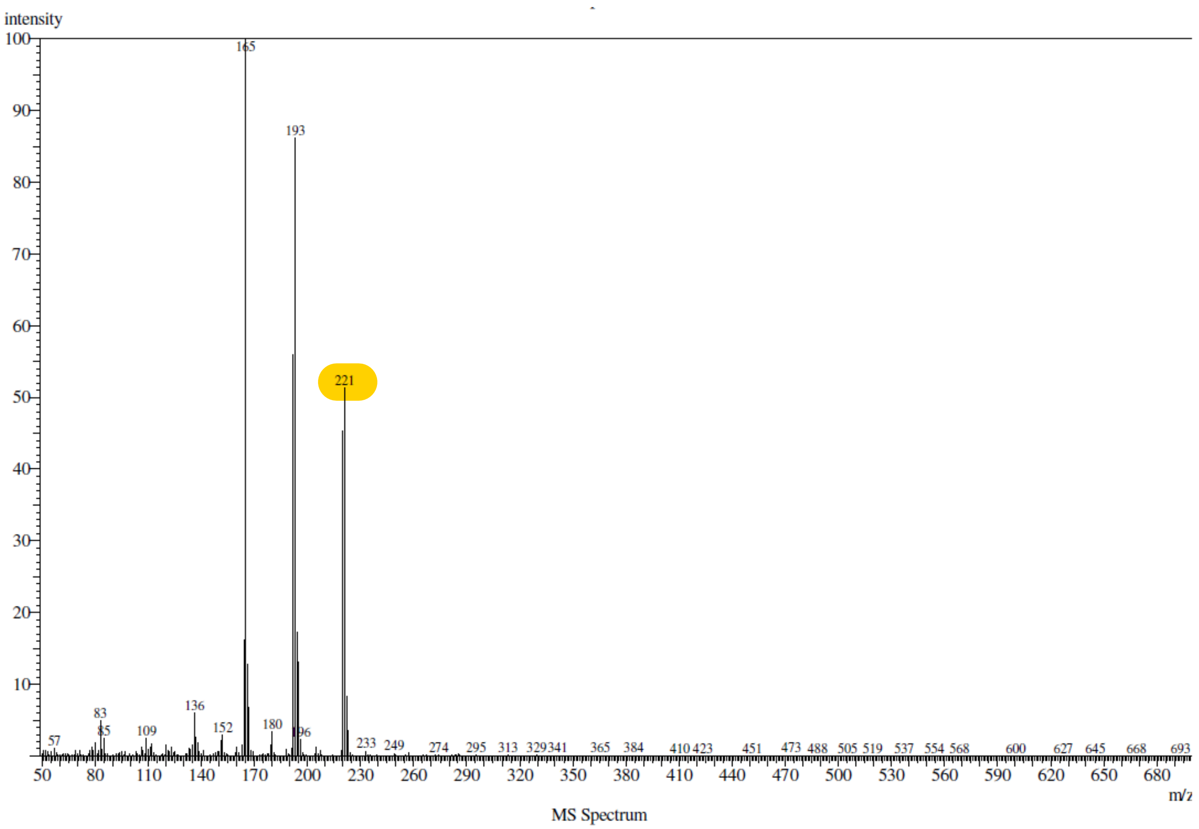

4. Antibacterial Activity

4.1. Minimum Inhibitory Concentration

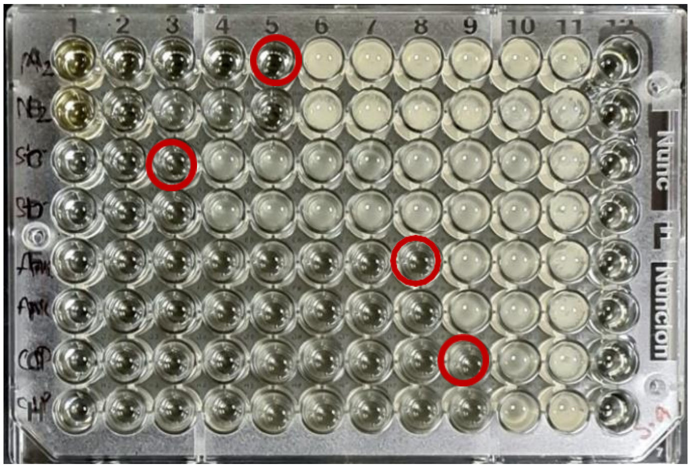

(a) *Staphylococcus aureus*

(2d) MIC- 8µg/mL

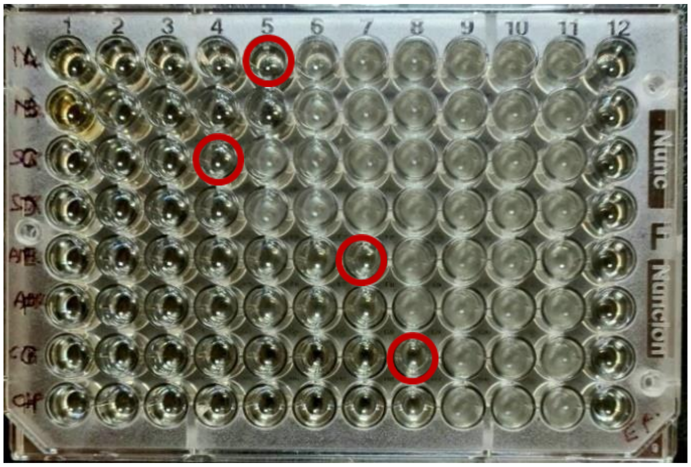

(b) *Enterococcus faecalis*

(2d) MIC- 8 µg/mL

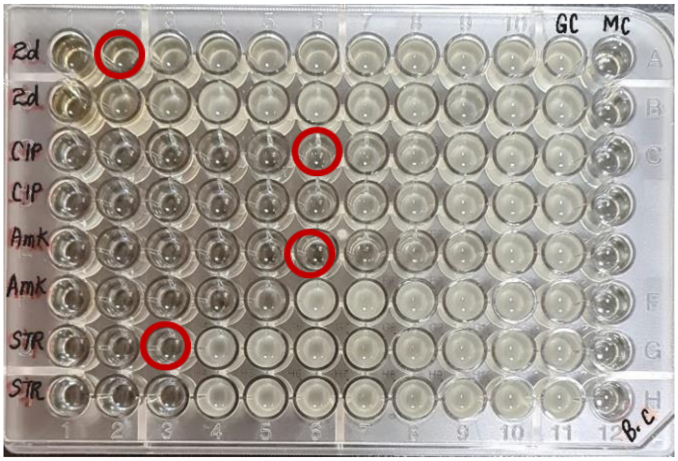

(d) *Bacillus cereus*

(2d) MIC - 64µg/mL

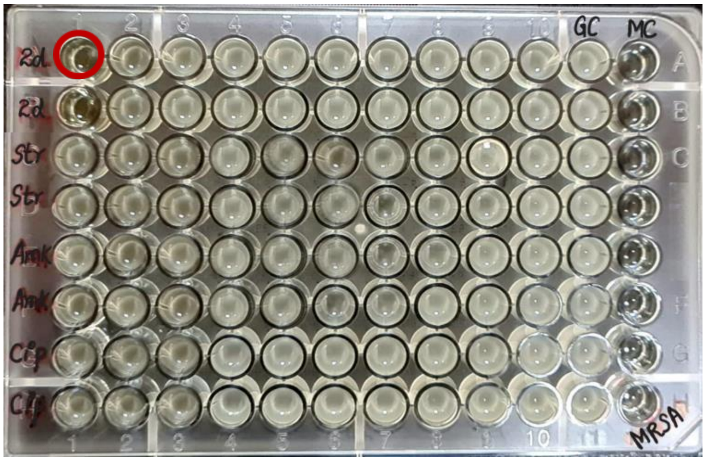

(d) *Methicillin* Resistant *S. aureus*

(2d) MIC - 128 µg/mL

FIGURE 1

Minimum Inhibitory Concentration of **2d**, STR, AMK and CIP against Gram- positive bacteria, (a) *Staphylococcus aureus*, (b) *Enterococcus faecalis*, (c) *Bacillus cereus* and (d) Methicillin Resistant *Staphylococcus aureus*. CIP (Represented by red-circled wells showing no visible growth compared to Growth Control well).

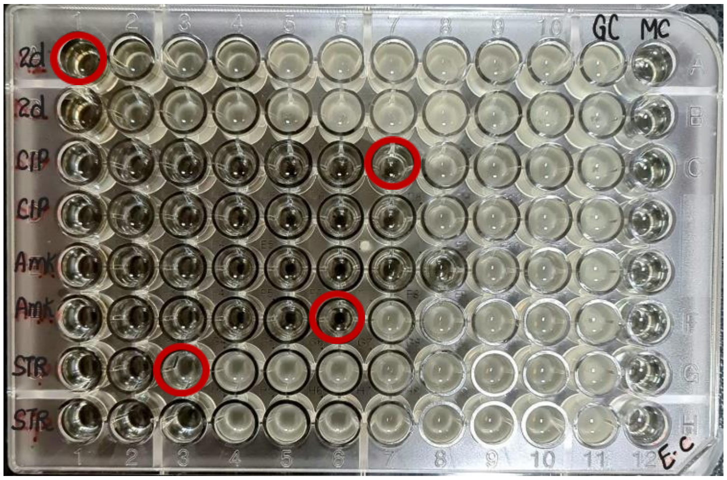

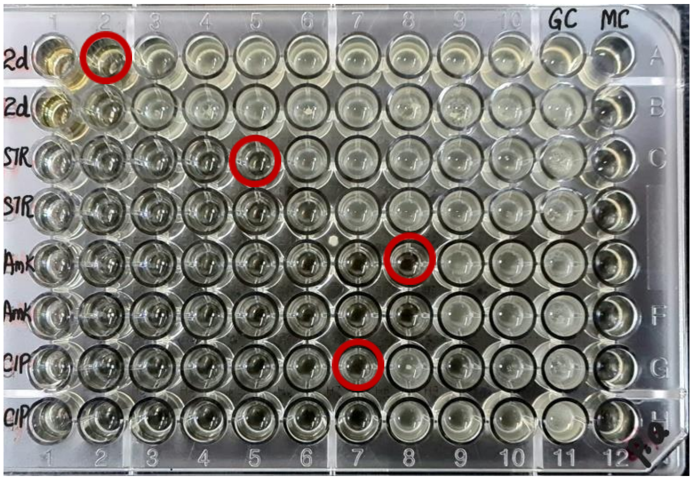

(a) ***Escherichia coli*** **(b)** ***Pseudomonas aeruginosa***

(2d) MIC- 128 µg/mL **(2d) MIC- 64 µg/mL**

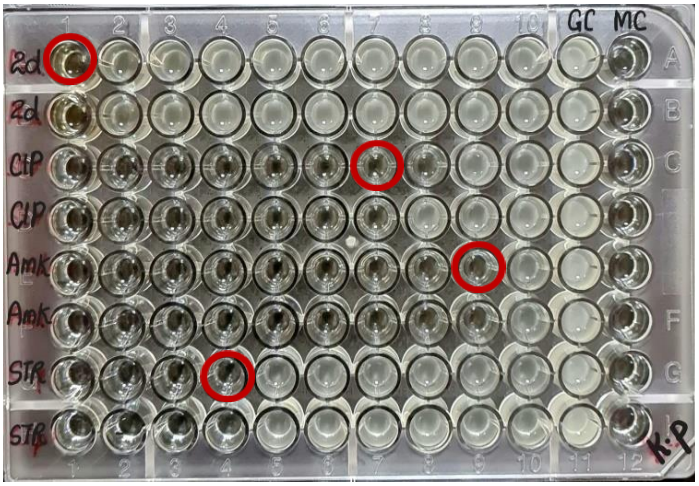

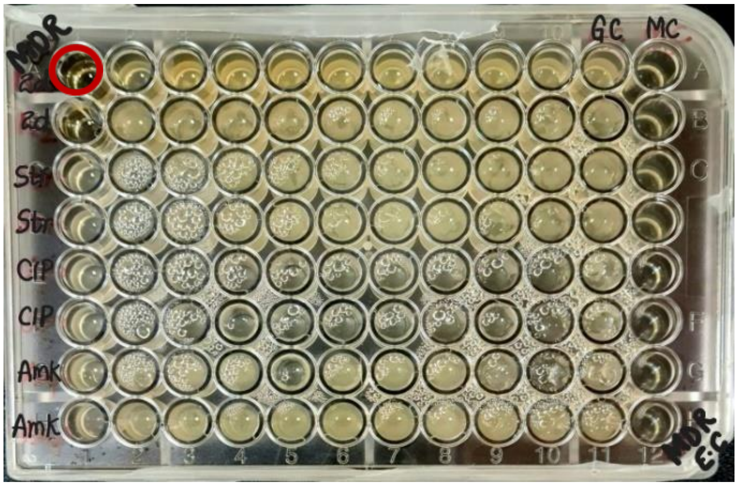

(c) ***Klebsiella pneumonia*** **(d) Multi-Drug Resistant *E. coli***

(2d) MIC- 128 µg/ml **(2d) MIC- 128 µg/ml**

FIGURE 2

Minimum Inhibitory Concentration of 2d, STR, AMK and CIP against Gram- negative bacteria, *Escherichia coli, Pseudomonas aeruginosa, Klebsiella pneumonia* and Multi-Drug Resistant *Escherichia coli*. (Represented by red-circled wells showing no visible growth compared to Growth Control well).

Percentage Inhibition of Growth

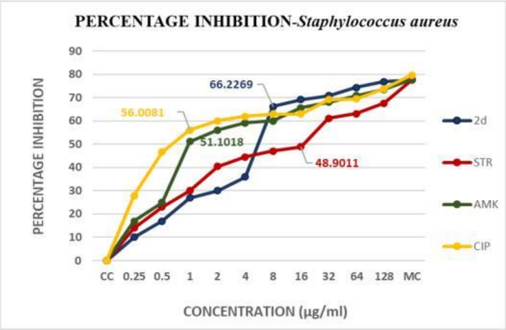

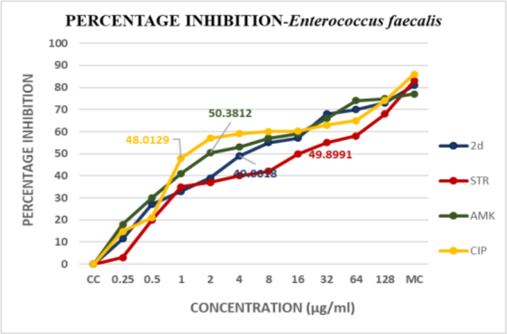

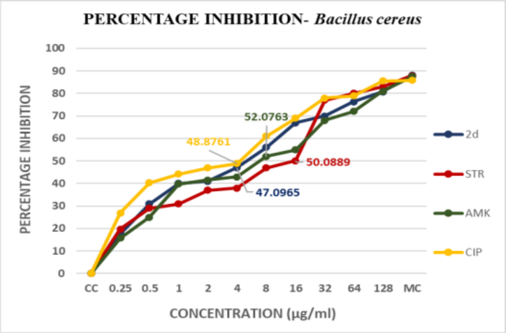

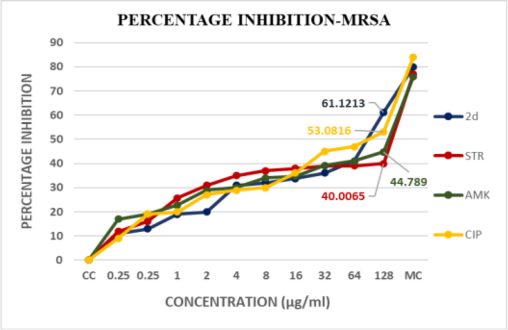

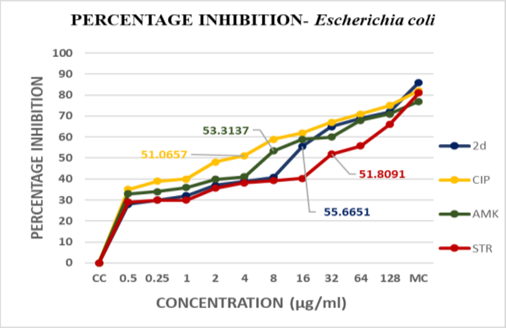

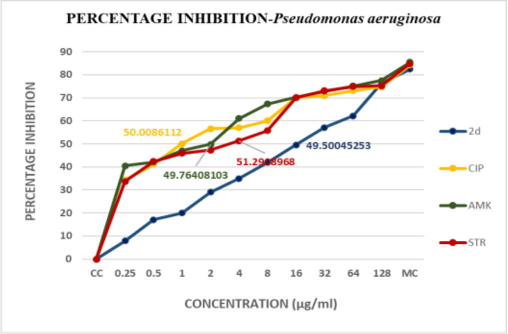

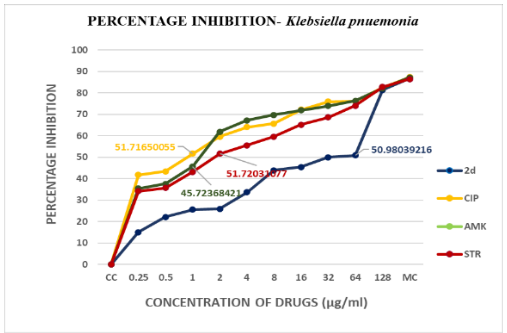

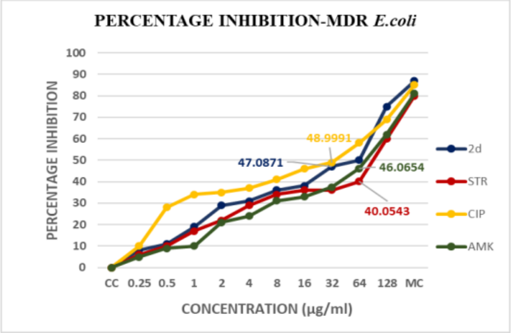

GRAPH 1

Spectrophotometric Analysis: Graphs showing Percentage Inhibition in Growth of Gram-Positive and Gram-Negative Bacteria in presence of Antibacterial compounds and concentrations at which 50% of the growth is inhibited by comparing with OD600 of Growth Control wells. (%) calculated by taking mean of 3 OD values.

4.2. Minimum Bactericidal Concentration

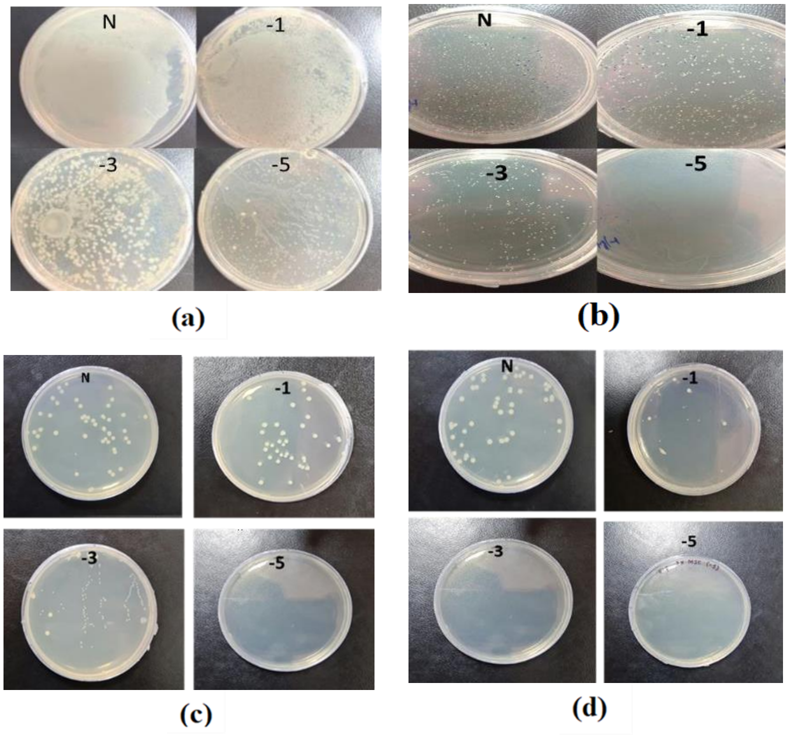

FIGURE 3

Result of CFU Enumeration of Enterococcus faecalis on Nutrient Agar plates after incubation at 37ºC for 24 hours (a) Untreated control plates (b) 2a treated plates (MIC) (c) 2b treated (MIC) (d) 2i (2X) MIC treated plates showing 99.9% Killing (more than 3 log reduction in CFU Count) Compared to untreated.

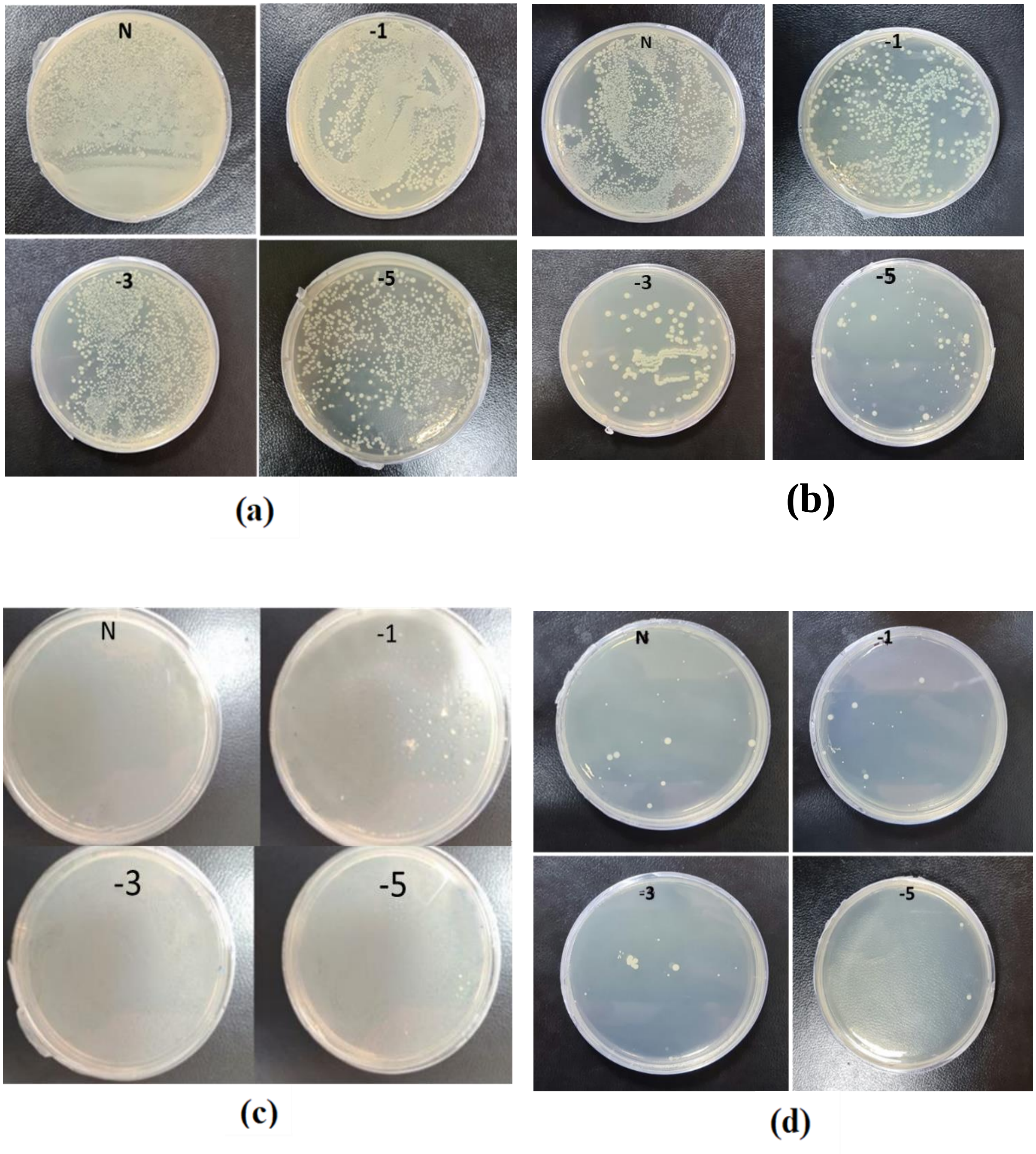

FIGURE 4

Result of CFU Enumeration as represented by circular colonies of Staphylococcus aureus on Nutrient Agar plates after incubation at 37ºC for 24 hours (a) Untreated control plates (b) 2d treated plates (MIC) (c) 2d treated plates showing 99.9% Killing (more than 3 log reduction in CFU Count) Compared to untreated (d) Ampicillin treated (2X) MIC.

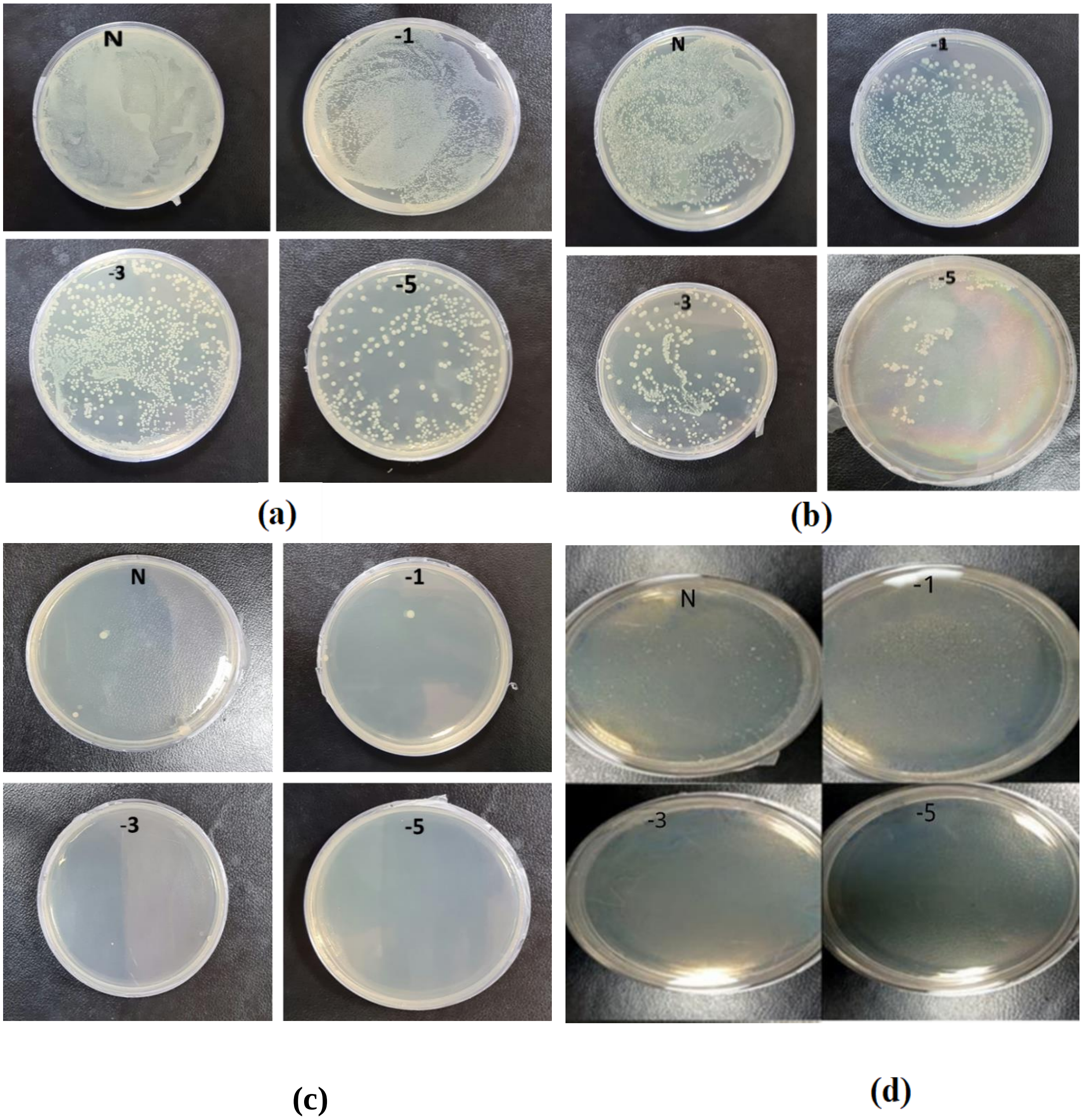

FIGURE 5

Result of CFU Enumeration as represented by circular colonies of Enterococcus faecalis on Nutrient Agar plates after incubation at 37ºC for 24 hours (a) Untreated control plates (b) 2d treated plates (MIC) (c) 2d treated plates showing 99.9% killing(more than 3 log reduction in CFU Count) Compared to untreated (d) Ampicillin treated (2X) MIC.

4.3. MTT Based Time Kill Kinetics

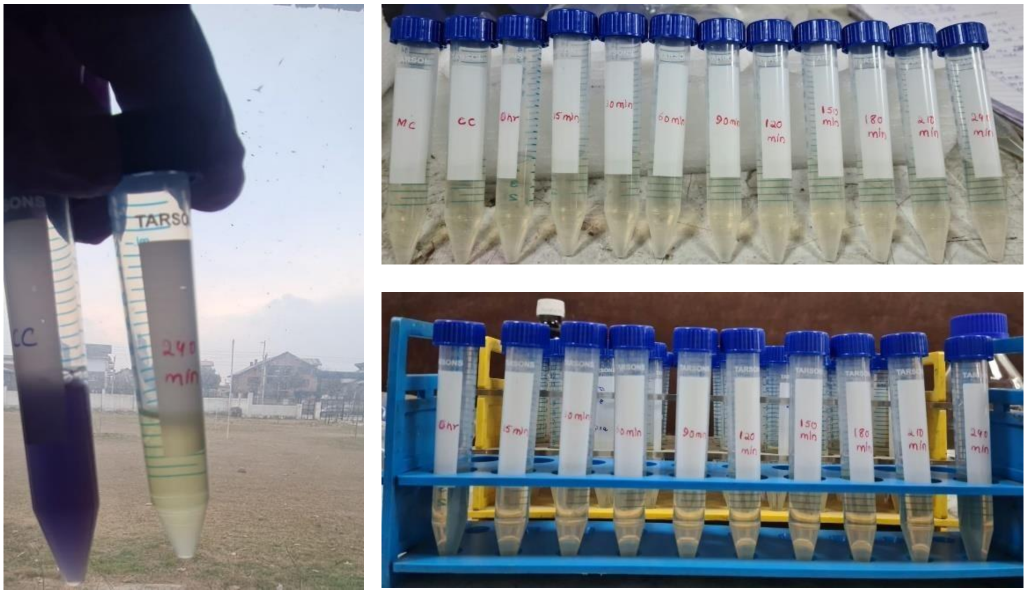

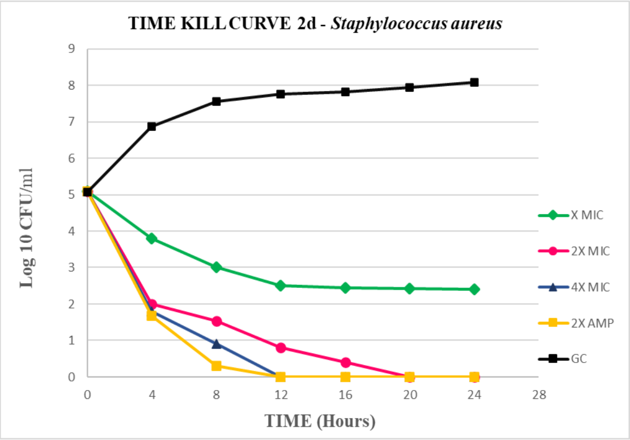

a

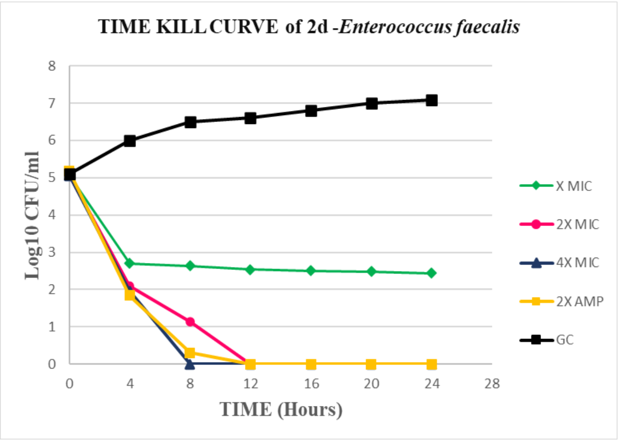

b

GRAPH 2

Time-kill curves of compound **2d**: The killing activity of **2d** against two bacteria was monitored for 24 hours by CFU Enumeration method. The compound concentration used in the experiment were MIC, 2x MIC, and 4x MIC. Ampicillin indicated by the yellow line was used as a positive control. For negative control (Black line), the cultures were incubated under the similar condition without any drug.

4.4. Biofilm Inhibition Assay

Table 1

Average Percentage of Biofilm Inhibition at Various Concentrations (Highlighted percentages indicate the percentage inhibition at MIC value of the compound **2d**).

**Percentage of Biofilm Inhibition at Concentration 0.25-128 µg/ml of Compound 2d**						
**Conc.**	*S. aureus*	*E. faecalis*	*B. cereus*	*E. coli*	*P. aeruginosa*	*K. pneumonia*
**128**	70.30	73.04	61.23	49.41	62.07	45.59
**64**	67.66	72.40	53.09	47.59	60.56	34.62
**32**	60.15	70.99	28.02	45.43	54.71	30.92
**16**	53.51	60.59	24.36	43.61	53.96	25.37
**8**	50.18	58.27	20.17	40.46	50.18	22.22
**4**	46.99	55.19	19.17	33.16	48.30	18.33
**2**	44.30	52.75	18.57	32.00	46.79	17.21
**1**	39.47	50.70	18.09	29.02	42.83	16.51
**0.5**	36.27	48.65	17.62	25.53	40.56	15.74
**0.25**	30.82	44.54	17.13	19.73	35.47	14.07
**GC**	0	0	0	0	0	0

4.5. Cytotoxicity Assay

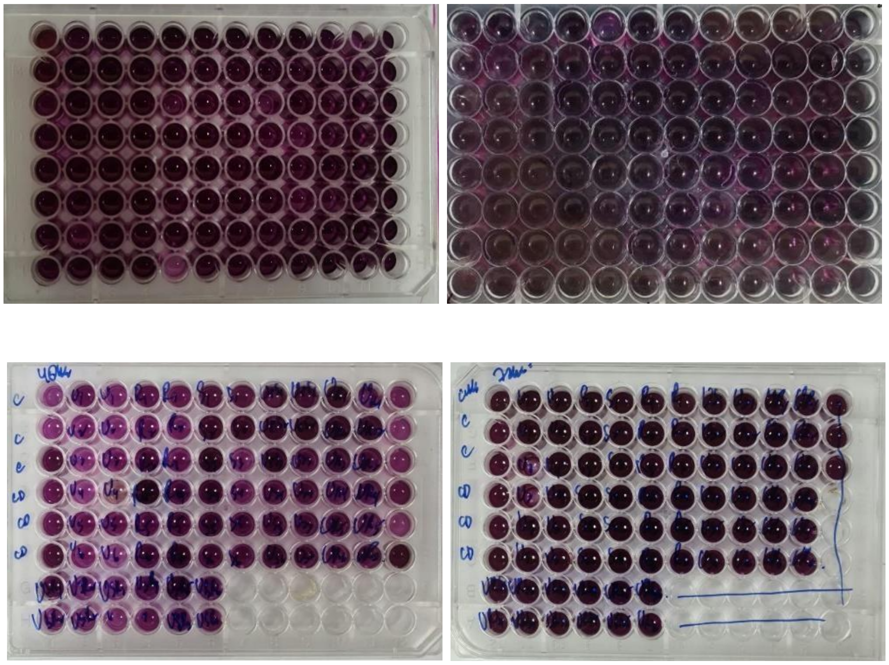

FIGURE 6

96-well plate showing Cell lines with treated different concentrations of **2d** for different time points showing color change on addition of MTT (due to reduction of MTT to formazan crystals) by viable cells indicating non-toxic effect of compound even at higher concentrations.
